# BR-bodies facilitate adaptive responses and survival during copper stress in *Caulobacter crescentus*

**DOI:** 10.1016/j.jbc.2025.110648

**Published:** 2025-08-28

**Authors:** Christie Passos, Dylan T. Tomares, Hadi Yassine, Wade E. Schnorr, Hannah Hunter, Helena K. Wolfe-Feichter, James Velier, Kathryn G. Dzurik, Julia Grillo, Alisa Gega, Sunil Saxena, Jared M. Schrader, W. Seth Childers

**Affiliations:** 1 Department of Chemistry, University of Pittsburgh, Pittsburgh, Pennsylvania, USA; 2 Department of Biological Sciences, Wayne State University, Detroit, Michigan, USA; 3 Department of Biology, Indiana University, Bloomington, Indiana, USA

**Keywords:** stress response, protein assembly, ribonuclease, copper, electron paramagnetic resonance, biomolecular condensate, RNase E, PNPase, *Caulobacter crescentus*, fluorescence, polyphosphate

## Abstract

Microbes must rapidly adapt to environmental stresses, including toxic heavy metals like copper, by sensing and mitigating their harmful effects. Here, we demonstrate that the phase separation properties of bacterial ribonucleoprotein bodies (BR-bodies) enhance *Caulobacter crescentus* fitness under copper stress. To uncover the underlying mechanism, we identified two key interactions between copper and the central scaffold of BR-bodies, ribonuclease E. First, biochemical assays and fluorescence microscopy experiments show that reduction of Cu^2+^ leads to cysteine oxidation, driving the transition of BR-bodies into more solid-like condensates. Second, tryptophan fluorescence and electron paramagnetic resonance assays reveal that ribonuclease E binds Cu^2+^ at histidine sites, creating a protective microenvironment that prevents mismetallation and preserves polynucleotide phosphorylase activity. In addition, we found that a substantial fraction of BR-bodies colocalizes with polyphosphate, which is also known to bind copper and facilitate the copper stress response. More broadly, this example illustrates how metal–condensate interactions can regulate the properties of condensate material and establish specialized chemical environments that protect enzyme function.

Biomolecular condensates, formed through phase separation, are dynamic, membraneless compartments that spatially organize biochemical reactions in both eukaryotes and bacteria (1, 2). In bacteria, which lack membrane-bound organelles, condensates play roles in RNA processing (3, 4, 5, 6, 7, 8), signal transduction (9, 10, 11, 12), chromosome segregation (13, 14), and many other processes (15, 16, 17, 18, 19, 20).

One of the defining features of condensates is their ability to form and dissolve in response to environmental stress (21). For example, yeast species have adapted to diverse thermal environments, revealing that while the formation of these condensates is evolutionarily conserved, their properties are fine-tuned to align with each species' unique ecological niches (22, 23, 24). Similar stress-responsive condensates are also essential for survival in bacteria (21), such as in the context of phosphate starvation (9) (3, 4, 25) and nitrogen starvation (26). Here, we further investigate whether bacterial ribonucleoprotein bodies (BR-bodies) protect bacteria during redox and heavy metal stress, thereby broadening our understanding of biomolecular condensates in microbial stress responses.

Ribonuclease E (RNase E) is a highly conserved bacterial enzyme essential for mRNA turnover. It is present in nearly half of all bacterial species (27). In *Caulobacter crescentus*, the RNA degradosome, anchored by the endoribonuclease RNase E as a scaffold, forms dynamic phase–separated condensates termed BR-bodies (3, 4, 25). RNase E, an endoribonuclease, performs the critical initial cleavage in RNA degradation and serves as a scaffold *via* its C-terminal intrinsically disordered region (3, 4, 28, 29, 30, 31, 32, 33, 34, 35, 36). BR-body phase separation is stimulated when untranslated mRNAs form weak multivalent interactions with RNase E and bring together RNase E, the 3′-5′ exoribonuclease polynucleotide phosphorylase (PNPase), and the helicase RhlB, accelerating RNA decay. Prior work has shown that BR-body formation is highly sensitive to changes in RNA abundance (25).

In *C. crescentus*, RNase E (CcRNase E) has an architecture comprising a structured N-terminal domain (NTD) responsible for catalysis and an intrinsically disordered C-terminal domain (CTD), which functions as a scaffold and facilitates phase separation (Fig. 1*A*). The CTD consists of intrinsically disordered regions with alternating positively and negatively charged patches. This arrangement enables the CTD to participate in various weak multivalent homotypic and heterotypic interactions through its interactions with RNA clients. Computational analysis using CIDER examining the CTD (residues 566–898) highlights its charge distribution (Fig. 1*B*) (37). Moreover, an AlphaFold2 structural model of CcRNase E (residues 1–898) is shown and color-coded to differentiate each region (Fig. 1*C*) (38). The structured NTD (residues 1–457) appears in *gray*, the putative Zn-binding domain (residues 458–471) in *purple* with cysteine residues highlighted in *yellow*, the dimerizing small domain in *green* (residues 472–565), and the disordered CTD in alternating *red* and *blue* (residues 566–898). In *Escherichia coli*, RNase E's Zn-link motif stabilizes its oligomeric structure by coordinating a single zinc ion through conserved cysteine residues. It is essential for catalytic activity but is not directly involved in catalysis. Disruption of the Zn-link causes loss of zinc, oligomer destabilization, and inactivation, while artificially maintaining a tetramer preserves activity, emphasizing the motif's structural role in organizing the active site (39).

**Figure 1:**
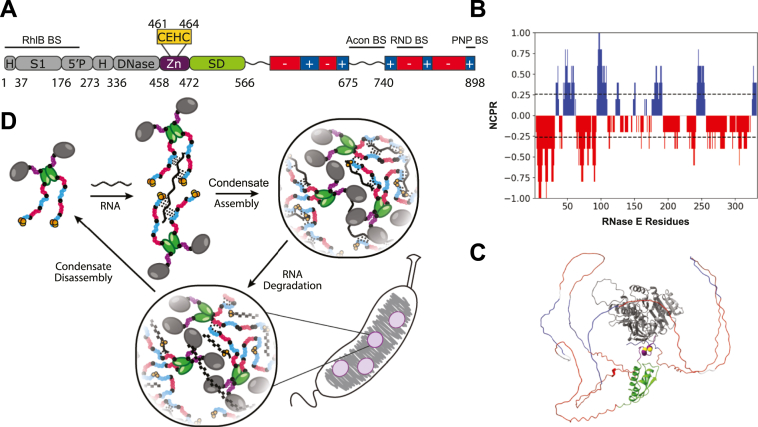
**Ribonuclease E (RNase E) is the central scaffold of bacterial ribonucleoprotein bodies (BR-bodies) that mediates global RNA decay in *Caulobacter crescentus*.***A*, domain architecture of RNase E. RNase E consists of four domains: the catalytic N-terminal domain (*gray*, 1–457), the zinc-binding domain (*purple*, with cysteines highlighted in *yellow*, 458–471), the dimerizing small domain (*green*, 472–565), and the disordered C-terminal domain (*red* and *blue*, 566–898). *B*, a CIDER analysis of RNase E’s IDR reveals a unique linear net charge per residue (NPCR) distribution as discrete alternating charge blocks within the C-terminal domain of RNase E(566–898) (38). *C*, an AlphaFold prediction of *C. crescentus* RNase E(1–898) is shown (38). *D*, RNase E serves as the central scaffold of the RNA degradosome and is the essential driver of BR-body phase separation. When the degradosome encounters untranslated mRNA, RNA degradosomes make multivalent interactions with mRNA that trigger phase separation. Mass action effects within the RNase E biomolecular condensate accelerate mRNA degradation and ultimately lead to dissolution of the BR-body upon degradation of the RNAs. IDR, intrinsically disordered region.

Importantly, deletion of the disordered CTD of RNase E, which mediates phase separation, reduces cell viability under a variety of stresses (3). This suggests that BR-bodies provide enhanced fitness during select stresses. For example, when *C. crescentus* cells were exposed to various acute stresses (*e.g.*, ethanol, EDTA, and heat shock), the stress response included an increased number of RNase E-YFP BR-bodies per cell and an increase in the intensity of the BR-bodies. As previously described by Al-Husini *et al.* (3), under no-stress conditions, an average of 1.1 RNase E-YFP BR-bodies per cell were observed to have formed. In comparison, the presence of 5 mM EDTA for 30 min promoted the formation of 2.0 BR-bodies per cell, whereas 10% ethanol for 30 min resulted in the formation of 2.1 BR-bodies per cell and a 5 min 42 °C heat shock produced 2.0 BR-bodies per cell (3). These findings suggest that BR-bodies not only act as dynamic RNA decay centers (Fig. 1*D*) (3, 25) but also as adaptive structures that respond to cellular stresses. However, it remains unclear whether these condensates respond to oxidative or heavy metal stress.

Copper is a particularly relevant environmental stressor that microbes experience. While essential as a cofactor, copper is highly toxic at high levels because of its redox activity, which can lead to mismetallation of proteins, disruption of Fe–S clusters, and reactive oxygen species production. *C. crescentus* encounters fluctuating copper concentrations in freshwater and soil environments and has evolved efflux-based detoxification strategies (PcoA/PcoB) and copper chemotaxis mediated by McpR (40, 41, 42). Here, we have examined the role of BR-body phase separation in facilitating *the survival of C. crescentus* under copper stress. We interrogate the functional interactions of copper and RNase E through a combination of *in vivo* and *in vitro* assays. These studies collectively suggest a model in which BR-bodies provide associated enzymes a protective zone from mismetallation inhibition.

## Results

### CuSO_4_ stress promotes reversible BR-body dissolution in *C. crescentus*

**Figure 2:**
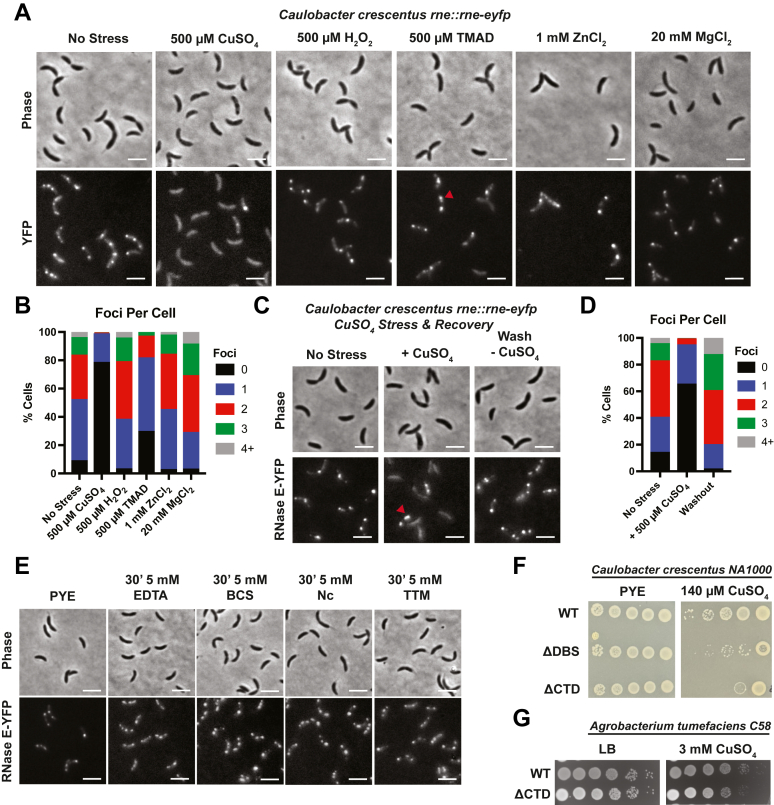
**BR-bodies are responsive and provide enhanced fitness when exposed to copper stress.***A*, representative phase contrast and fluorescence microscopy imaging of *Caulobacter crescentus* expressing RNase E(1–898)-eYFP from its endogenous promoter (JS51, *rne::rne-eYFP*) in the absence and presence of either 500 μM CuSO_4_, 500 μM H_2_O_2_, 500 μM TMAD, 1 mM ZnCl_2_, or 20 mM MgCl_2_ for 8 min. The scale bar denotes 3 μm. *B*, quantification of the percentage of BR-bodies per cell in *C. crescentus* expressing RNase E-eYFP in the absence (n = 5947) (8 biological replicates) or presence of either 500 μM CuSO_4_ (n = 2289) (2 biological replicates), 500 μM H_2_O_2_ (n = 1613) (2 biological replicates), 500 μM TMAD (n = 1522) (3 biological replicates), 1 mM ZnCl_2_ (n = 957) (3 biological replicates), or 20 mM MgCl_2_ (n = 2285) (2 biological replicates). Amongst stresses, CuSO_4_ selectively leads to dissolution of BR-bodies. *C*, representative phase contrast and fluorescence microscopy imaging of a CuSO_4_ washout experiment with *C. crescentus* expressing RNase E-eYFP from its endogenous promoter (JS51). After CuSO_4_ exposure, cells were washed in fresh medium to remove CuSO_4_ and then reimaged to observe the recovery of RNase E-eYFP foci after 30 min. We observe recovery of foci after removal of copper from the media. *D*, quantification of the number of BR-bodies per cell pre-CuSO_4_ exposure (n = 870), during CuSO_4_ exposure (n = 1182), and after washout (n = 754) (2 biological replicates). *E*, representative phase contrast and fluorescence microscopy imaging of *C. crescentus* expressing RNase E-eYFP from its endogenous promoter in the absence and presence of 5 mM broad-spectrum EDTA metal chelator *versus* 5 mM specific copper chelators, bathocuproine sulfonate (BCS) disodium salt hydrate, neocuproine (Nc), and ammonium tetrathiomolybdate (TTM) for 30 min. Chelation of metals leads to an increase in BR-body intensity (2 biological replicates). *F*, recruitment of RNase E clients into BR-bodies and BR-body phase separation provide enhanced fitness under copper stress. Efficiency of plating assay with wildtype *C. crescentus* RNase E, degradosome-binding site mutant (ΔDBS) RNase E, and NTD C-terminal deletion mutant (ΔCTD) RNase E in the absence and presence of 130 μM CuSO_4_. *Red triangle* in *A* and *C* points to static and elongated BR-bodies in TMAD and CuSO_4_ stress (three biological replicates). *G*, efficiency of plating assay with wildtype *Agrobacterium tumefaciens C58* RNase E and the RNase E NTD C-terminal deletion mutant (ΔCTD) RNase E in the absence and presence of 3 mM CuSO_4_ (three biological replicates). BR-body, bacterial ribonucleoprotein body; CTD, C-terminal domain; NTD, N-terminal domain; RNase E, ribonuclease E; TMAD, tetramethylazodicarboxamide.

Previous studies have shown that ethanol and EDTA stress enhance BR-body formation (3), prompting us to investigate whether BR-body assembly responds to oxidative and metal stress. In addition, past studies by Luisi *et al.* (39) have identified that *E. coli* RNase E has a CxxC motif that binds Zn^2+^ ions. Based on these past observations, we investigated the impact of metals and redox stress to determine their effect on BR-body formation (Fig. 2, *A* and *B*).

Under no stress conditions, we observed an average of 1.6 ± 0.3 RNase E-eYFP foci/cell in *C. crescentus* cells (Figs. 2*B* and S1, *A* and *B*). In comparison, when cells were exposed to CuSO_4_ stress for 8 min (Fig. 2*A*), cells displayed a more diffuse subcellular localization pattern with considerably fewer BR-bodies by 500 μM CuSO_4_ with an average of 0.24 ± 0.5 foci/cell (Figs. 2, *A*, *B* and S1, *A* and *B*). Notably, about 20% of cells contained BR-bodies that were larger and more intense than in the no-stress condition. We next considered whether the CuSO_4_-mediated reduction in foci resulted from oxidative stress by examining the effects of *N*,*N*,*N*′,*N*′-tetramethylazodicarboxamide (TMAD), also known as diamide, and H_2_O_2_ treatment (Figs. 2, *A*, *B* and S1, *C*–*F*). We found that adding 500 μM TMAD led to a decrease to 0.92 ± 0.8 foci/cell (Fig. S1, *C* and *D*). In addition, a small population of cells exposed to TMAD formed larger and brighter BR-bodies than those observed in the control condition without stress. However, increasing amounts of TMAD from 600 μM to 1 mM were unable to fully dissolve the condensates, as observed with CuSO_4_ addition (Figs. 2*B* and S1, *C* and *D*). We found that exposure to H_2_O_2_ for 8 min had little impact on subcellular localization, with an average of 1.85 ± 0.9 foci/cell (Figs. 2, *A*, *B* and S1, *E* and *F*). This suggests that the dissolution of BR-bodies by CuSO_4_ may be a specific effect of copper, as opposed to a generalized oxidative stress effect.

Next, we considered that CuSO_4_ might have a nonspecific effect on the subcellular localization of other biomolecular condensates. Thus, we examined an additional protein that phase separates in *C. crescentus*, PopZ, a cell-cycle signaling scaffolding protein that localizes to the cell poles (43, 44). Unlike the impact on RNase E’s subcellular localization, we found that 500 μM CuSO_4_ did not affect the subcellular localization of PopZ-mCherry condensates (Fig. S1*G*). This suggests that CuSO_4_ specifically affects BR-bodies, either directly or indirectly.

Biomolecular condensates are characterized by their capacity to undergo rapid and reversible changes in formation and dissolution upon changes in stress (21). We thus considered whether BR-body formation would recover after removing CuSO_4_ stress (Fig. 2, *C* and *D*). Before adding copper, we observed 1.69 ± 1.1 foci/cell, but after exposure to 500 μM CuSO_4_ for 8 min, the number of foci per cell decreased to 0.41 ± 0.6. Interestingly, amongst the 33.9% that display one or two foci, there exists a population of cells with larger and more elongated BR-bodies than those observed in the no-stress condition (Fig. 2*D*, *red triangle*). After washing cells in fresh medium for over 30 min to remove CuSO_4_, we observed complete recovery of BR-bodies, with cells displaying 2.39 ± 1.2 foci/cell (Fig. 2, *C* and *D*). Interestingly, analysis of average total cell intensity revealed a 54.4% decrease upon the addition of CuSO_4_ (Fig. S1*H*). This suggests that copper binding to RNase E may cause localized quenching of YFP, potentially reducing our ability to detect BR-bodies. In addition, it is possible that the intensity decrease results from RNase E degradation. Overall, our findings demonstrate that CuSO_4_ stress induces a reversible and RNase E-specific effect on RNase E subcellular localization in *C. crescentus* cells.

Having previously observed the ability of CuSO_4_ to dissolve BR-bodies at short times (8 min) (Fig. 2, *A* and *B*), we examined the impact of CuSO_4_ at an extended 1-h exposure (Fig. S2*A*). After a 1-h exposure to CuSO_4_, *C. crescentus* cells exhibited an increase in RNase E-eYFP total cell intensity (Fig. S2*B*). This suggests that prolonged CuSO_4_ exposure may influence RNase E protein expression levels or protein degradation. Notably, the 5ʹ-UTR of the *rne* transcript is a substrate of BR-bodies (25), and copper-induced changes in BR-body phase separation could alter RNA half-life, potentially affecting protein expression. In addition, extended exposure may allow cellular responses to mitigate cytoplasmic copper levels through reduction mechanisms (40, 41, 45, 46).

### Copper-specific chelators lead to increased BR-body intensity

Given the acute impact of copper exposure on the dissolution of BR-bodies (Fig. 2, *A* and *B*) and the increase in BR-body intensity when exposed to EDTA (Fig. 2*E* and S2*C*), we wondered if these phenomena are connected. EDTA exhibits a strong preference for chelating metal ions, including calcium (Ca^2+^), magnesium (Mg^2+^), and copper (Cu^2+^), along with other transition metals, such as iron (Fe^2+^/Fe^3+^), lead (Pb^2+^), and zinc (Zn^2+^) (Fig. S2*A*) (47). We observed that EDTA treatment led to an increase in BR-body intensity, rising from 972 ± 257.81 arbitrary units (AUs) (no stress) (n = 2803) to 1297 ± 291.45 AU (n = 1272) (*p* < 0.001) (Fig. S2*C*), consistent with our previous findings (3).

To test if more specific metal chelation could be sufficient, we compared the effects of EDTA with three copper-specific chelators: bathocuproine sulfonate (BCS), neocuproine (Nc), and ammonium tetrathiomolybdate (TTM) (Fig. 2*E*). BCS preferentially binds Cu^1+^, whereas TTM selectively binds Cu^2+^ (Fig. S2*C*). In comparison, Nc more promiscuously interacts with various metals, including Cu^1+^, Fe^2+^, and Zn^2+^ (Fig. S2*C*). We found that these copper-specific chelators increased BR-body intensity compared with the no-stress condition. In the absence of stress, we measured an intensity of 972 ± 257.81 AU (n = 2803), whereas the intensities for the copper chelators were 1383 ± 370.75 AU (BCS, *p* < 0.001) (n = 1876), 1544 ± 310.63 AU (TTM, *p* < 0.001) (n = 1775), and 1337 ± 297.92 AU (Nc, *p* < 0.001) (n = 2265) (Fig. S2*C*).

Since copper-specific chelators increase BR-body intensity, we wondered whether the BR-bodies could sequester copper under conditions with low metal content. Such a material response would share similarities to the observation of enhanced BR-bodies during phosphate nutrient limitations (4). To test this hypothesis, we examined whether the RNase E’s ability to recruit clients and undergo phase separation enhances fitness during metal chelation (Fig. S2*D*). We performed an efficiency of plating (EOP) assay to compare the growth of wildtype *C. crescentus* with strains expressing either the RNase E NTD alone or a mutant lacking the ΔCTD under copper-specific chelation. The EOP results (Fig. S2*D*) indicate that when cells are grown under EDTA chelation, Nc and TTM strains lacking the capacity to form BR-bodies demonstrated reduced fitness. In comparison, cells expressing full-length RNase E or the RNase E degradosome-binding site (DBS) mutant displayed robust growth in the presence of the tested chelators. These findings suggest that under metal-depleted conditions, the RNase E condensate environment may help facilitate client binding to essential metal cofactors. In addition, prolonged metal depletion stress on solid media may induce changes in RNase *E* transcription that promote survival.

### BR-body formation provides high fitness during copper-induced stress

Given the impact of copper stress on the subcellular localization of RNase E-eYFP, we investigated whether these biochemical processes are critical for cell fitness. To address this, we examined the capacity of *C. crescentus* to survive copper stress using an EOP assay (Figs. 2*F* and S2*E*). We observed colony growth in wildtype strains at CuSO_4_ concentrations as high as 200 μM (Fig. S2*E*). This level of copper toxicity aligns with findings from Matroule *et al*. (40), who reported a substantial decrease in the cumulative mass of *C. crescentus* (41) under similar CuSO_4_ stress conditions in peptone–yeast extract (PYE) (41). Next, we investigated whether RNase E's phase separation capacity and client recruitment functions contribute to the copper stress response in *C. crescentus*. We constructed a *C. crescentus* strain that expressed only the NTD (residues 1–577) of RNase E, which neither phase separates nor recruits client proteins (JS769). To differentiate the effects of client recruitment from phase separation, we utilized a strain with deletions of the binding sites for PNPase, aconitase, and RNase D (RNase E ΔDBS, JS801). Previous studies have shown that the RNase E ΔDBS mutant can phase separate into BR-bodies but does not recruit PNPase, RNase D, or aconitase (3).

EOP assays conducted in the presence of 130 μM CuSO_4_ revealed that wildtype strains exhibit enhanced fitness compared with RNase E ΔDBS strains (Fig. 2*F*). Similarly, we examined the fitness of *Agrobacterium tumefaciens* in LB supplemented with 3 mM CuSO_4_, comparing the wildtype strain to a strain expressing RNase E ΔCTD as the sole variant (Figs. 2*G* and S2*F*). We found that *Agrobacterium* expressing an RNase E variant that is incapable of stimulating phase separation as a sole copy exhibited reduced cell fitness. These results collectively suggest that the protective role of the BR-body phase-separated environment is also conserved in Agrobacterium.

Moreover, these results suggest that the recruitment of PNPase, aconitase, RNase D, and potentially other client proteins into BR-bodies may be essential for maintaining high fitness under copper stress. This may be because BR-bodies provide a protective zone that retains the functional capabilities of client proteins. Alternatively, the client proteins themselves may play some role in attenuating the toxicity of the copper metals. In addition, the removal of the client binding sites may impact the recruitment of the other 111 client proteins of BR-bodies (48).

In contrast, the RNase E NTD variant, which also lacks the capability for phase separation, demonstrated even lower fitness in the presence of CuSO_4_. This suggests that BR-body phase separation capacity plays a crucial role in *C. crescentus*' response and enhanced fitness during copper stress. Interestingly, despite copper’s general toxicity to *C. crescentus*, in the presence of copper, the mRNA half-life changes little upon copper exposure (*i.e.*, 0.52 min, whereas at 125 μM CuSO_4,_ the half-life was 0.79 min, and at 500 μM, the half-life was 0.46 min, as shown in Figs. S3*A* and S3*B*). This supports the idea that the degradosome function is insensitive to CuSO_4_ up to 125 μM.

### Polyphosphate partially colocalizes with BR-bodies in *C. crescentus* and may contribute to protection from copper stress

To further investigate how BR-bodies might contribute to copper tolerance beyond mRNA degradation, we turned our attention to polyphosphate, a known mediator of metal detoxification in bacteria and a potential interacting partner of the BR-body network. Polyphosphate contributes to bacterial copper tolerance by chelating Cu^2+^ ions (49) and facilitating their export. In *E. coli*, this process requires polyP synthesis by PPK, degradation by PPX, and export *via* PitA/B symporters, which cotransport phosphate and metal ions (50). Similar mechanisms are conserved across diverse bacterial species, indicating a broad role for polyphosphate in mitigating metal stress. Given the association between polyphosphate biosynthetic enzyme PPK1 and RNase E observed in *E. coli* (51), and given the importance of BR-bodies in *C. crescentus* during Cu(II) stress adaptation (Fig. 2), we asked whether polyphosphate granules colocalize with BR-bodies and whether this interaction could contribute to the enhanced stress tolerance conferred by BR-bodies (52, 53).

**Figure 3:**
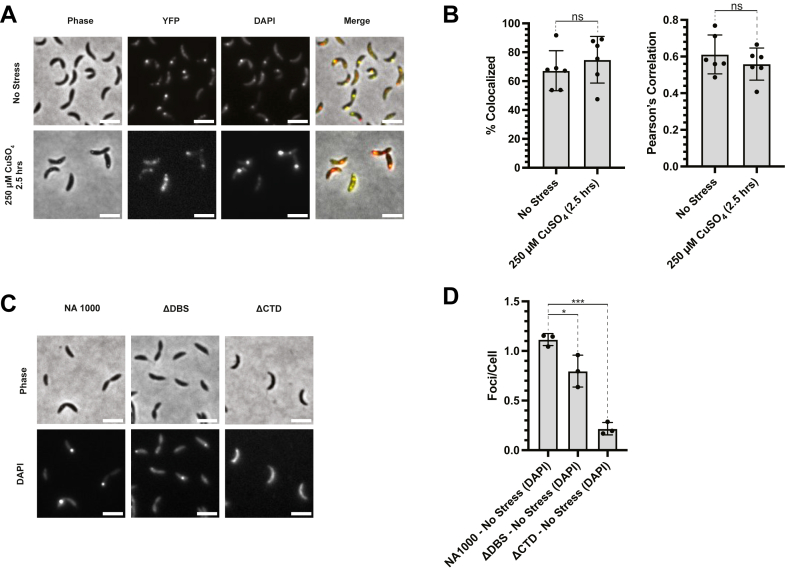
**Polyphosphate partially colocalizes with BR-bodies in *Caulobacter crescentus* and may contribute to protection from copper stress.***A*, representative phase contrast, RNase E-YFP, DAPI, and merged stained polyphosphate granule fluorescence images of wildtype *C. crescentus* (*rne::rne-eyfp*) cells under control and copper stress conditions. The scale bar denotes 3 μm. Fig. S4 contains the extended set of conditions for Figure 3*A*. A set of images labeled “no stress” are reused in Fig. S4 to enable direct comparison with the supplementary dataset. *B*, colocalization analysis of BR-bodies and DAPI-stained polyphosphate granules shows that 67.24% of control cells under no stress conditions exhibit overlap between RNase E-YFP and polyphosphate foci. Pearson's correlation analysis quantifying the spatial colocalization between RNase E-YFP and DAPI-stained polyphosphate granules reveals that copper stress does not significantly enhance their colocalization (six biological replicates). *C*, representative phase contrast and DAPI images of wildtype, ΔDBS (degradosome-binding site), and ΔCTD (C-terminal domain) *rne* mutants under copper stress. The scale bar denotes 3 μm. *D*, quantification of DAPI-stained polyphosphate foci per cell shows a substantial reduction in polyphosphate granules in strains lacking BR-bodies, suggesting a role for BR-body formation in promoting or stabilizing polyphosphate accumulation during stress (three biological replicates). BR-body, bacterial ribonucleoprotein body; DAPI, 4′,6-diamidino-2-phenylindole; RNase E, ribonuclease E.

To address this, we imaged wildtype *C. crescentus* (*rne::rne-eyfp*) cells expressing RNase E-YFP under control and copper stress conditions (Fig. 3*A* and S4*A*). RNase E-YFP formed distinct foci consistent with BR-body formation. Under imaging conditions that preferentially stain polyphosphate–4′,6-diamidino-2-phenylindole (DAPI) complexes in *C. crescentus*, we also observed punctate polyphosphate granules (54). Colocalization analysis revealed that a substantial fraction of RNase E-YFP foci overlapped with DAPI-stained polyphosphate granules, with 67.2% of control cells exhibiting colocalization (Figs. 3*B* and S4*B*). To determine whether copper exposure enhances this interaction, we quantified colocalization under both conditions and found no significant difference, suggesting that polyphosphate granules are constitutively associated with BR-bodies, regardless of copper stress. While not all BR-bodies are colocalized with polyphosphate, the heterogeneous localization is similar to that observed for Hfq and RhlE with RNase E (48).

To test whether BR-bodies influence the biosynthesis, formation, or stability of polyphosphate granules, we examined RNase E mutants deficient in BR-body formation (Fig. 3*C*). In the ΔDBS mutant, which lacks the DBS and fails to recruit client proteins, such as PNPase, aconitase, and RNase D, and the ΔCTD mutant, which is defective in BR-body formation and client protein binding, we observed a reduction in the number of DAPI-stained polyphosphate foci per cell (Fig. 3*D*). These results suggest that BR-body formation may promote polyphosphate polymerization, stabilization, or accumulation within BR-bodies (49). Moreover, the substantial loss of fitness in copper stress that occurs with the RNase E ΔCTD mutant may be at least in part because of loss of robust polyphosphate granules.

Together, these findings show that BR-bodies are spatially associated with polyphosphate granules and may play a role in maintaining polyphosphate levels during copper stress. Given the established role of polyphosphate in copper detoxification and its association with BR-bodies, it may contribute to the enhanced fitness of *C. crescentus* under Cu(II) stress conditions (52, 53, 55).

### The CxxC motif contains sequence variation in the flanking residues

**Figure 4:**
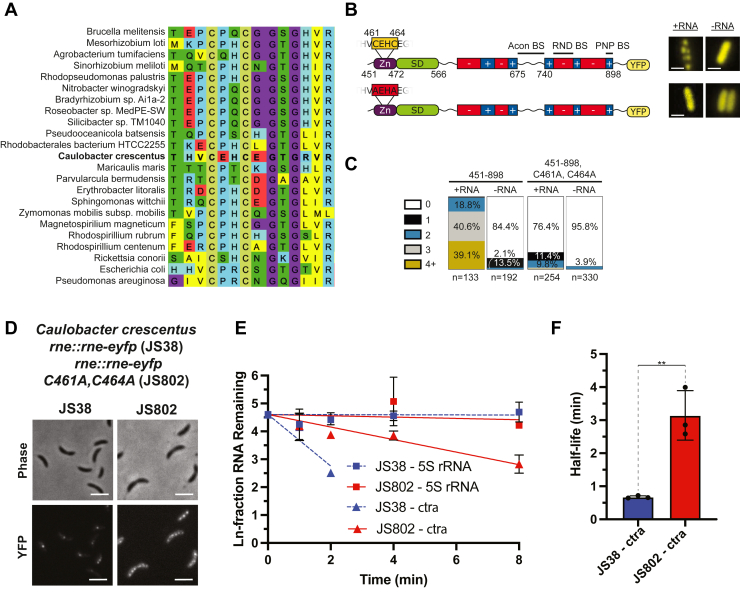
**RNase E CxxC motif plays a critical role in BR-body assembly and ribonuclease function *in vivo*.***A*, the CxxC motif is conserved throughout α-proteobacterial RNase E homologs. *B*, comparison of RNase E(451–898)-eYFP, RNase E(451–8987, C461A)-eYFP, RNase E(451–898, C464A)-eYFP, and RNase E(451–898, C464A, C461A)-eYFP when heterologously expressed in *Escherichia coli* demonstrates that at least one cysteine is necessary for foci formation. *C*, the quantification of foci in the panel above supports an increase in the number of foci/cell when at least one cysteine is present. *D*, representative phase contrast and fluorescence microscopy imaging of *Caulobacter crescentus* expressing RNase E(451–898)-eYFP from its endogenous promoter (JS8) and *C. crescentus* RNase E(451–898), C461A, C464A)-eYFP variant. The scale bar denotes 3 μm. Three biological replicates were performed. *E*, comparison of RNA half-life for 5S rRNA and ctrA for wildtype RNase E *versus* mutant RNase E CTD C464A C461A (three biological replicates). *F*, quantification of mRNA half-life for CtrA for wildtype RNase E *versus* mutant RNase E CTD C464A C461A (p = 0.005) (three biological replicates). BR-body, bacterial ribonucleoprotein body; RNase E, ribonuclease E.

Given the observed effects of CuSO_4_ on BR-body formation and its role in supporting high fitness during copper stress, we aimed to investigate if the conserved CxxC motif in the annotated Zn-link was sensitive to the addition of copper. Past biochemical studies of *E. coli* RNase E provide evidence for a conserved CxxC motif that mediates tetramerization in a Zn^2+^-dependent manner of *E. coli* RNase E (39). The CxxC motif is well conserved across the α-proteobacteria (CPHC, Fig. 4*A*) and in the γ-proteobacteria (CPRC). The consensus sequences of the α-proteobacteria (CPHC) and γ-proteobacteria (CPRC) are highly similar, both containing a proline in the second position. Proline induces β-turns and can specifically impart rigidity to CxxC motifs if placed in the second or third position. Uniquely, *C. crescentus* RNase E has lost that proline in its second position (CEHC, Fig. 4*A*), which could suggest it is more flexible. The next neighboring amino acid of *E. coli* RNase E (CPRCS) also differs from that of *C. crescentus* (CEHCE). Overall, the introduction of glutamates and the replacement of arginine for histidine may alter the function of the CxxC motif. The CxxC motif is also known to oxidize to generate disulfide bonds. The bacterial thioredoxin *Bacillus subtilis* ResA utilizes a CxxC motif in a loop region that can form disulfides. Therefore, the *C. crescentus* RNase E CxxC motif may be readily oxidized to form disulfide bonds.

### *C. crescentus* RNase E C461 and C464 contribute to phase separation *in vivo*

First, we considered the importance of the cysteines to phase separation of RNase E *in vivo* through heterologous expression in *E. coli* of RNase E variants designed as single and double mutations of the conserved cysteines (C461 and C464) to alanine (Fig. 4*B*) within CTD (residues 451–898), which is sufficient for phase separation of RNase E (3). A single mutation of either cysteine (C461 or C464) to alanine did not abolish phase separation (C461A: 98.0% localized, n = 409; C464A: 98.6% localized, n = 362), and cells displayed RNA-responsive foci. However, the double cysteine to alanine mutant displayed a 76.4% diffuse protein population (n = 254), suggesting that functional cysteines are necessary for RNase E(451–898)-eYFP foci formation in *E. coli* (Fig. 4, *B* and *C*).

Our findings demonstrate that at least one cysteine is required for the phase separation of heterologously expressed CcRNase E in *E. coli* (Fig. 4, *B* and *C*), which prompted us to explore whether this also applies to CcRNase E when expressed in *C. crescentus*. To test this, we generated a CcRNase E C461A/C464A variant lacking both cysteines and assessed its ability to form foci (JS802) (Fig. 4*D*). Interestingly, the results show that this variant can still form foci and even display an increased average number of foci per cell compared with the *rne::rne-eyfp* control (Fig. S4*A*). Specifically, the wildtype RNase E-eYFP displays an average of 1.2 ± 0.6 foci/cell (n = 473), whereas the C461A/C464A variant shows 1.8 ± 1.3 foci/cell (n = 354), a 28% increase (Fig. S3*C*). Notably, as the C461A/C464A variant is expressed, the unlabeled wildtype is depleted in this strain. So, a small amount of wildtype RNase E may influence the subcellular localization of the C461A/C464A variant.

When examining partition ratios (the average focus intensity over background), the wildtype control has a significantly higher average partition ratio (4.24 ± 0.28, n = 473) than the C461A/C464A variant (2.81 ± 0.30, n = 354) (Fig. S3*C*). This suggests that the absence of cysteines may lead to more diffuse or less tightly packed protein localization. The increased localization of RNase E in *C. crescentus* could be influenced by the inclusion of the NTD, which may enhance valency, cognate RNAs present in the cellular milieu, or specific clients in *C. crescentus* that promote phase separation of the C461A/C464A variant. Nonetheless, the data indicate that the RNase E C461A/C464A variant exhibits altered subcellular localization. In addition, we observed that in the presence of the RNase E C461A/C464A variant, the ctrA mRNA half-life was substantially increased to 3.14 min *versus* 0.67 min for wildtype (Fig. 4, *E* and *F*). This is consistent with variants’ impact on the phase separation functions of the CTD, as well as the roles of the cysteines in *E. coli* RNase E’s ribonuclease domain reported by Luisi *et al.* (39).

### Cu^2+^ facilitates the formation of disulfide bonds in RNase E

**Figure 5:**
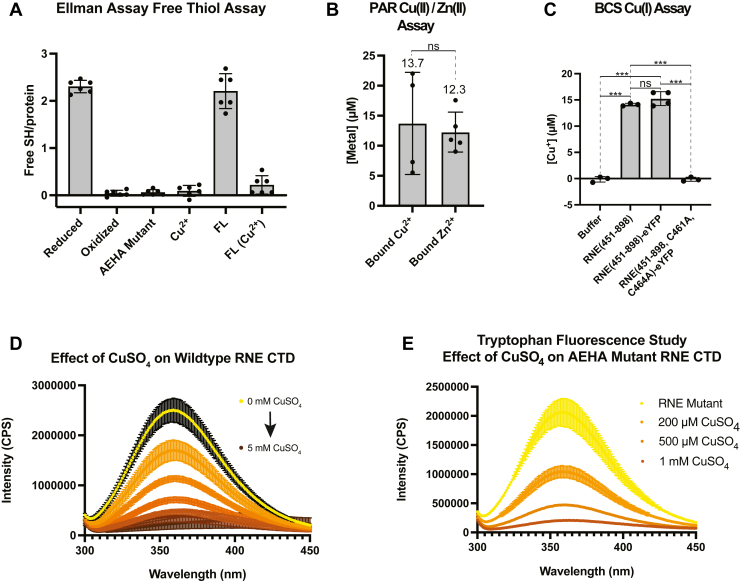
**Copper interacts with RNase E through two distinct mechanisms: Cu(II) mediates disulfide bond formation and, in addition, binds in a cysteine-independent binding mode.***A*, an Ellman assay reveals a copper-specific interaction that limits C461- and C464-free thiol access. RNase E does not bind zinc *in vitro*, but free thiol access is sensitive to disulfide bond formation. *B*, PAR Cu(II)/Zn(II) assay provides further evidence for Cu(II) binding to RNase E. Error was determined based on four technical replicates and two biological replicates for Cu^2+^ and five technical replicates and two biological replicates for Zn^2+^. *C*, schematic representation of bathocuproine sulfonate (BCS) coordinating with Cu^1+^ in a BCS assay. Quantification of Cu^1+^ produced by reduction of Cu^2+^, upon exposure to unlabeled RNase E(451–898) and RNase E(451–898)-eYFP, which consist of both cysteines, *versus* RNase E(451–898, C461A, C464A)-eYFP in which both cysteines have been mutated to alanines. Quantification reveals that the concentration of Cu^1+^ substantially increases in the presence of both cysteines (*p* < 0.001). Error was determined based on three technical replicates for the buffer-only condition, three technical replicates for RNE(451–898), four technical replicates for RNE(451–898)-eYFP, and three technical replicates for RNE(451–898, C461A, C464A)-eYFP. *D*, unlabeled wildtype RNase E(451–898) titration by Cu^2+^ (*top* to *bottom*: 0 μM, 50 μM, 100 μM, 200 μM, 400 μM, 700 μM, 1 mM, 1.5 mM, and 5 mM CuSO_4_) monitored by protein intrinsic fluorescence intensity quenching with a plot of fluorescence intensity *versus* CuSO_4_ concentration. Increasing concentrations of CuSO_4_ result in a decrease in tryptophan fluorescence intensity. Error was determined based on two biological replicates and three technical replicates. *E*, unlabeled RNase E(451–898, C461A, C464A) variant titration by Cu^2+^ monitored by protein intrinsic fluorescence intensity quenching. Increasing concentrations of CuSO_4_ result in a decrease in tryptophan fluorescence intensity. Error was determined based on two biological replicates and two technical replicates. PAR, 4-(2-pyridylazo)resorcinol; RNase E, ribonuclease E.

We next examined if the CxxC motif that binds Zn^2+^ ions (39) may be sensitive to oxidation by Cu^2+^ stress. We, therefore, used the Ellman assay to measure the number of free thiol groups in the presence and absence of copper and oxidation conditions (Figs. 5*A* and S5*C*). The assay revealed significant loss of free thiols upon addition of Cu^2+^ (0.1 ± 0.1, *p* < 0.0001) (Figs. 5*A* and S5*C*), indicating that C461 and C464 may function as a Cu-mediated sensor in *C. crescentus*.

We hypothesize that Cu^2+^ may directly bind to RNase E or facilitate cysteine oxidation. To test the direct binding model, we performed a 4-(2-pyridylazo)resorcinol (PAR) assay, which confirmed that an average of 13.7 μM Cu^2+^ bound to RNase E when the protein was incubated with a total of 50 μM CuSO_4_ (Figs. 5*B* and S5*D*). Similarly, we observed that 12.3 μM Zn^2+^ bound to RNase E when the protein was incubated with a total of 50 μM ZnSO_4_ (Figs. 5*B* and S5*D*). If Cu^2+^ facilitates the formation of disulfide bonds (Fig. S5*A*), it should generate Cu^1+^, which can be quantified using the colorimetric chelator BCS. BCS binds Cu^1+^ and exhibits strong absorption at 483 nm. In addition, BCS does not interact with Cu^2+^, allowing for the selective measurement of Cu^1+^. A standard curve using BCS and Cu^1+^ under reducing conditions was constructed to quantify the amount of Cu^1+^ (Fig. S5*B*).

To determine whether RNase E reduces Cu^2+^ to Cu^1+^, we desalted freshly reduced *Cc*RNase E into a buffer lacking reducing agents and incubated 10 μM RNase E with BCS following a 2-min incubation with a 10-fold excess of Cu^2+^. This reaction yielded 15.2 ± 1.5 μM Cu^1+^, whereas the control reaction without protein showed no detectable Cu^1+^ formation (0.3 ± 0.4 μM) (Figs. 5*C* and S5*E*). To confirm the role of cysteines in Cu^2+^ reduction, we tested RNase E(451–898, C461A, C464A)-eYFP under the same conditions. This variant produced no appreciable Cu^1+^ (−0.1 ± 0.9 μM) (Figs. 5*C* and S5*E*), suggesting that Cu^2+^ induces disulfide bond formation in RNase E. Together with the Ellman assay data, these results indicate that Cu^2+^ binding to RNase E leads to its reduction to Cu^1+^, concomitant with RNase E disulfide bond formation.

Building on the Ellman and BCS assay results, which revealed that Cu^2+^ induces disulfide bond formation in RNase E (Fig. 5, *A* and *C*), we next explored whether the disulfide bonds were intermolecular, leading to an increased oligomerization state. We analyzed RNase E(451–898) and RNase E(1–898, D403C) incubated with varying amounts of Cu^2+^, using native PAGE (Fig. S5, *F* and *G*) and RNase E(451–898) with nonreducing SDS-PAGE (Fig. S5*H*). If intermolecular disulfide bonds were formed, we would expect tetramers under native conditions. However, no appreciable change in the oligomerization state was detected under native conditions, indicating that intermolecular disulfide bonds were absent. Thus, the observed reduction in free thiol content is likely because of the formation of intramolecular disulfide bonds.

### Cu^2+^ can interact directly with RNase E and does not require C461 or C464

Experiments at this stage suggest that Cu^2+^ binds to RNase E and can facilitate disulfide bond formation. This leads to the key question of whether Cu^2+^ can coordinate cysteines or other nearby metal-binding residues. We, therefore, examined the quenching of the intrinsic tryptophan fluorescence intensity of the protein as a function of increasing concentrations of CuSO_4_. The unlabeled RNase E (50 μM) was incubated in a buffer of 100 mM NaCl and 20 mM Tris–Cl, pH 7.5, with and without varying concentrations of CuSO_4_. Decreasing fluorescence intensity was correlated with increasing concentrations of CuSO_4_ for unlabeled wildtype RNase E(451–898) (Figs. 5*D* and S5*I*) and the unlabeled C461A/C464A variant (Figs. 5*E* and S5*J*). These data suggest that Cu^2+^ can interact with RNase E at alternative residues other than C461 or C464. Notably, these assays were performed within a 20 mM Tris buffer, which may compete with the protein for binding to copper. However, assays performed in the Mops buffer confirmed a similar decrease in tryptophan fluorescence intensity, suggesting Tris does not interfere with RNase E–Cu(II) interaction (Fig. S6, *A* and *B*). In addition, after observing that Cu^2+^ leads to a reduction in tryptophan fluorescence, we added EDTA and found that the tryptophan fluorescence reversibly returned (Fig. S6*C*). These tryptophan fluorescence assays indicated that Cu(II) binds sufficiently close to tryptophan to facilitate quenching, or that Cu(II) induces conformational changes in RNase E, altering the local chemical environment around tryptophan residues and thereby reducing fluorescence intensity. Moreover, these assays suggest that Cu(II) binds to RNase E in a C461/C464-independent manner.

**Figure 6:**
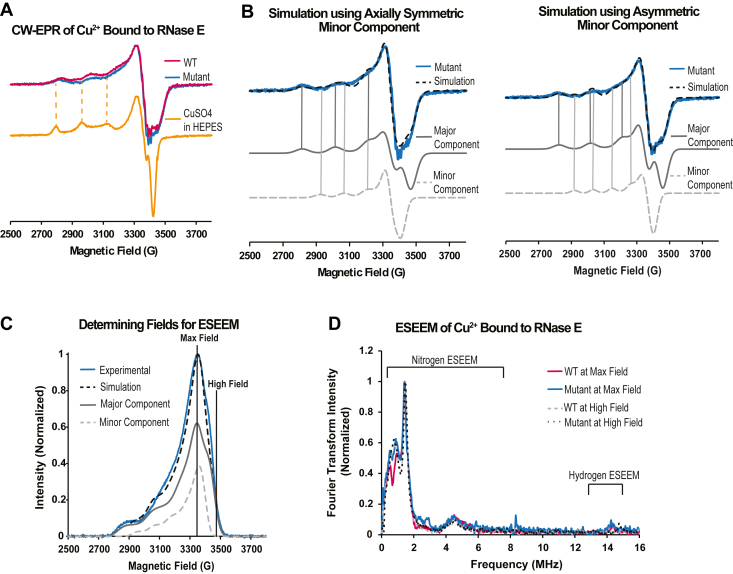
**EPR provides support that RNase E binds to Cu^2+^ independent of cysteines and in association with the imidazole ring of a histidine.***A*, CW-EPR spectra, collected at 80 K, of RNase E(451–898) wildtype (*pink*), the RNase E(451–898, C461A/C464A) variant (*blue*), and CuSO_4_ in Hepes buffer, pH 7.5 (*yellow*). The *dashed yellow lines* highlight the absence of peaks because of Cu^2+^ coordination to Hepes buffer in the protein samples. As the peak positions and intensities are almost identical in both RNase E samples, only the C461A/C464A variant data are shown in *B*. CW-EPR spectra of RNase E wildtype data can be found in the Supporting Information. *B*, simulation of the C461A/C464A variant (*blue*) is overlaid in *dashed black*. The RNase E spectrum shows two components. The major component (*gray*) accounts for 70% of the simulation (*dashed black*), and the minor component (*dashed light gray*) accounts for the remaining 30% of the simulation (*dashed black*). The minor component was simulated using both axially symmetric (*left*) and asymmetric (*right*) coordination geometry. *C*, field-swept spectrum of the RNase E C461A/C464A mutant (*blue*) overlaid with the integrated CW-EPR simulation (*dashed black*) using an asymmetric coordination geometry for the minor component (*dashed light gray)*. ESEEM experiments were carried out at the field with the maximum intensity (3340 G) and a higher field (3463 G), highlighted with *black lines*. At the high field, only the major component (*gray*) contributes to the spectrum. *D*, ESEEM spectra of RNase E wildtype and C461A, C464A mutant collected at the maximum intensity (*pink* and *blue*, respectively), RNase E wildtype collected at the high field (*dashed gray*), and RNase E mutant collected at the high field (*dotted black*). All spectra show peaks indicative of Cu^2+^ interacting with the imidazole ring of histidine. CW, continuous wave; EPR, electron paramagnetic resonance; ESEEM, electron spin-echo envelope modulation; RNase E, ribonuclease E.

To validate these tryptophan fluorescence quenching observations, we can directly investigate the interaction sites of Cu^2+^ using electron paramagnetic resonance (EPR) spectroscopy (56). The line shape of continuous wave (CW) EPR provides information on the identity of the atoms that are directly coordinated to Cu^2+^ (57). Notably, the spectrum of the RNase E wildtype and C461A/C464A mutant shows almost identical peak positions (Fig. 6*A*), indicating very similar Cu^2+^ binding in both protein samples. Note that the line shape is distinct from that of free CuSO_4_ in buffer, showing that all Cu^2+^ is bound to RNase E. Interestingly, of the 700 μM Cu^2+^ added to the sample, only 155 μM Cu^2+^ for RNase E wildtype and 306 μM Cu^2+^ for RNase E C461A/C464A mutant can be accounted for by EPR, suggesting that oxidation of the cysteines may generate Cu^1+^ species that are not visualizable by EPR.

Simulation of the data required two components (Figs. 6*B* and S7, *A* and *B*), which indicates two distinct Cu^2+^-binding sites on the protein. The major component (75% for wildtype and 70% for the mutant) in both samples indicates an octahedral, square planar, or square pyramidal metal-binding site (57, 58, 59, 60). The major components in both proteins had a g_‖_ of 2.218 and A_‖_ of 187 G. By comparing these values to previously reported values for different combinations of nitrogen, oxygen, and sulfur coordination, it becomes clear that the major component is due to nitrogen and oxygen coordination in the equatorial plane (57, 61, 62). In addition, because the major component has the exact same g_‖_ and A_‖_ values in the wildtype and C461A/C464A variant, cysteines are not involved in the Cu^2+^ coordination of the major component.

The minor component of the RNase E wildtype and C461A/C464A variant (25% and 30%, respectively) also exhibited the same g- and hyperfine parameters. However, a distinct coordination geometry was less clear. When simulating using axially symmetric tensors, we found a g_‖_ of 2.203 and A_‖_ of 136 G (Fig. 6*B*, *left*). These values do not align with expected values for an axially symmetric geometry, and A_‖_ values smaller than 140 G can point toward a trigonal planar or tetrahedral coordination (63, 64, 65, 66). Therefore, we also simulated the data using coordination for the minor component. This fit achieved a similar quality of fit to the data as the one that assumes an octahedral coordination (Fig. 6*B*, *right*).

To further probe the binding sites of Cu^2+^, we performed electron spin-echo envelope modulation (ESEEM) experiments. ESEEM can observe the interaction between the electron spin of Cu^2+^ and the nuclear spin of elements within 4 to 8 Å of Cu^2+^ (67, 68). As the simulation of the CW-EPR data identifies two components, we performed the ESEEM experiments at two different fields. The first field is at the maximum intensity of the field-swept spectrum and samples both the major and minor components (Fig. 6*C*). The second higher field samples only the major component. The resulting ESEEM spectra are shown (Fig. 6*D*). The field-swept spectrum for the RNase E wildtype and the raw time domain ESEEM spectra can be found in the Supporting Information (Fig. S7, *C* and *D*). Each spectrum shows three peaks below 2 MHz and one broad peak at 4 MHz, which indicates coordination to the imidazole ring of histidine (68, 69, 70, 71). Moreover, the integrated intensity of the peaks below 11 MHz compared with the integrated intensity of the peak at 14 MHz can be used to estimate the number of histidines that are coordinated to Cu^2+^ (47, 56, 57). Surprisingly, when measured at the maximum field, both the RNase E wildtype and the C461A/C464A variant show coordination to a single histidine. The spectra for both samples measured at the high field also show coordination to only one histidine. The CW-EPR and ESEEM data confirm that in the major component, Cu^2+^ is directly bound to one histidine and no cysteine. Interestingly, the data also indicate that the minor component is likely also bound to one histidine residue. Our data collectively indicate that Cu^2+^ can interact with RNase E in two distinct ways: Cu^2+^ facilitates disulfide bond formation^,^ and Cu^2+^ can coordinate directly with His residues.

### Copper solidifies RNase E condensates in a cysteine-dependent manner *in vitro*

**Figure 7:**
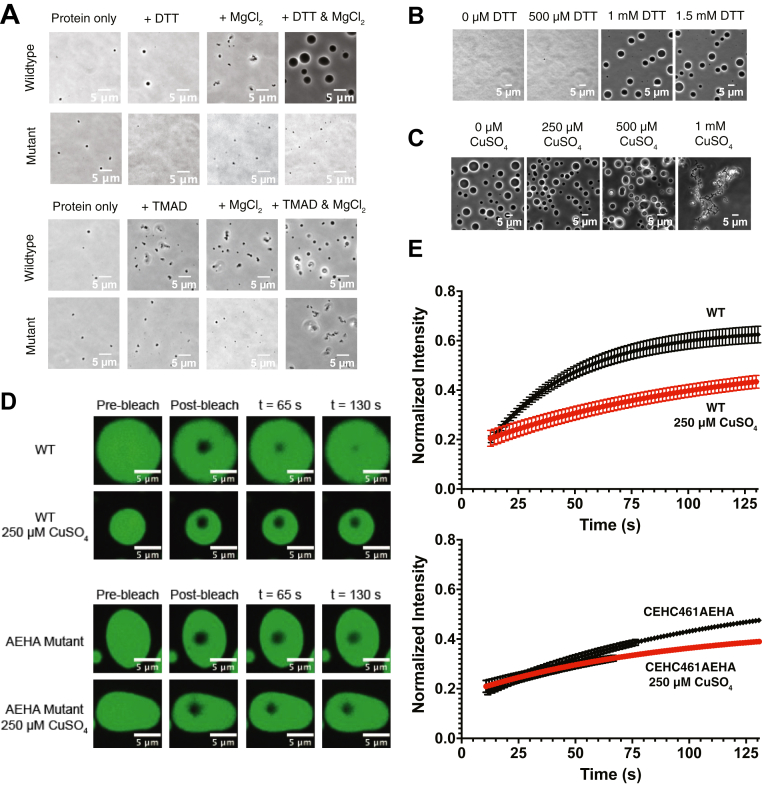
***In vitro* RNase E condensate material properties are regulated by copper binding and reducing conditions in a manner that is dependent upon C461 and C464.***A*, representative phase contrast microscopy imaging of unlabeled RNase E(451–898) reveals that RNase E phase separation with MgCl_2_ requires the presence of a reducing agent (DTT), whereas the presence of an oxidizing agent (TMAD) promotes aggregation. The scale bar denotes 5 μm. Error is based on two technical replicates and one biological replicate. *B*, representative phase contrast microscopy images of unlabeled RNase E(451–898) demonstrate that an increase in DTT concentration positively correlates with an increase in RNase E phase separation. The scale bar denotes 5 μm. Error is based on two technical and two biological replicates. *C*, representative phase contrast microscopy images of unlabeled RNase E(451–898) further demonstrate that high concentrations of CuSO_4_ promote RNase E aggregation. The *in vitro* droplet imaging assays consist of 20 μM RNase E incubated in 20 mM Tris buffer, pH 7.4, and 70 mM NaCl in the absence or presence of varying concentrations of DTT and CuSO_4_, 1 mM TMAD, and 20 mM MgCl_2_. The scale bar denotes 5 μm. Error is based on three technical replicates and two biological replicates. *D*–*E*, the presence of CuSO_4_ impacts RNase E material properties, causing RNase E droplets *in vitro* to become more solid like, resulting in slower recovery from fluorescence recovery after photobleaching (FRAP) for both wildtype and the C461A/C464A variant. The scale bar denotes 5 μm. Error is based on three technical replicates. RNase E, ribonuclease E; TMAD, tetramethylazodicarboxamide.

Given the significant role of RNase E’s cysteines in phase separation *in vivo* (Fig. 4), we investigated how these residues influence phase separation *in vitro*. We first assessed the effect of reducing conditions on the ability of RNase E to phase separate in the presence of MgCl_2_. To do so, we purified unlabeled wildtype CcRNase E(451–898) and conducted phase separation assays by mixing 20 μM protein to produce conditions of 70 mM NaCl and 20 mM Tris with either 1 mM DTT only, 20 mM MgCl_2_ only, or both 1 mM DTT and 20 mM MgCl_2_. We also evaluated the phase separation behavior of the RNase E(451–898, C461A/C464A) variant under the same conditions (Fig. 7*A*, *top*). Phase contrast microscopy revealed that wildtype CcRNase E(451–898) forms phase-separated droplets only in the presence of MgCl_2_ when reducing conditions are maintained. To optimize reducing conditions, a dose–response analysis in the presence of 20 mM MgCl_2_ revealed that concentrations of at least 1 mM DTT were necessary for robust phase separation of CcRNase E(451–898) (Fig. 7*B*).

In the absence of the reducing agent, freshly desalted RNase E(451–898), stored in 20 mM Tris, pH 7.5, and 200 mM NaCl, forms only small aggregates, even in the presence of MgCl_2_ (Fig. 7*A*, *top*). In contrast, the C461A/C464A variant did not form micron-scale–sized assemblies under any surveyed conditions, indicating the importance of cysteines and reducing conditions in the phase separation of RNase E. We further explored the effects of oxidizing conditions using 1 mM TMAD. Under these conditions, RNase E(451–898) formed smaller, dynamically arrested assemblies in the presence of MgCl_2_, suggesting that free cysteines are essential to the initiation and coarsening of RNase E assemblies (Fig. 7*A*, *bottom*).

Building on previous *in vivo* observations, where CuSO_4_ caused RNase E foci in *C. crescentus* cells to diffuse at high concentrations for short incubation times, we examined its impact on RNase E phase separation *in vitro*. We titrated increasing amounts of CuSO_4_ (0–1 mM) into preformed RNase E droplets to assess changes in droplet morphology (Fig. 7*C*). Imaging revealed that at concentrations above 500 μM, CuSO_4_ induced a dynamic arrest of RNase E droplets, resulting in aggregate-like assemblies.

To further characterize the effect of CuSO_4_ on the material properties of RNase E droplets, we measured fluorescence recovery after photobleaching (FRAP) of RNase E(451–898)-YFP assemblies. We examined whether CuSO_4_ causes wildtype RNase E(451–898)-YFP and the C461A/C464A variant to adopt a more solid-like state, as the aggregates observed at high CuSO_4_ concentrations suggested. FRAP analysis was conducted at 250 μM CuSO_4_ (Fig. 7*D*). Increasing CuSO_4_ concentrations resulted in slowed FRAP, indicating a transition to more solid-like RNase E assemblies (Fig. 7*E*).

We next considered whether this copper-stimulated change in RNase E material properties depended on C461 and C464. Unlike the wildtype RNase E protein, adding Cu^2+^ to RNase E(451–898, C461A/C464A)-YFP led to milder differences in the FRAP experiment. Moreover, using phase microscopy, we observed that wildtype RNase E(451–898), when combined with PNPase and poly(A) substrate, formed biomolecular condensates with diameters that ranged from 1.8 μm to 11.7 μm (n = 226) (Fig. S8*A*). In comparison, in the presence of PNPase and poly(A), the RNase E(451–898) C461A/C464A variant only formed small assemblies ranging from 0.5 to 1.8 μm in diameter (Fig. S8*B*). This supports a model in which Cu^2+^ interactions at a site distal from C461 to C464 cysteine motif may facilitate networking interactions separate from disulfide bond formation, resulting in more solid-like RNase E condensate assemblies.

### RNase E condensates maintain PNPase activity in the presence of CuSO_4_

**Figure 8:**
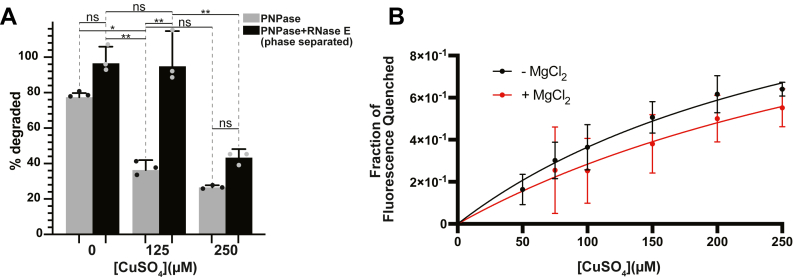
**RNase E condensates preserve the ribonuclease activity of PNPase when exposed to CuSO4 *in vitro.****A*, percent of poly(A) degraded in the presence of PNPase with or without wild RNase E(451–898) using a fluorescent thioflavin-T tracking endpoint assay. RNase E progressively shields PNPase from Cu^2+^ mismetallation as Cu^2+^ concentration increases. Error is based on three technical replicates. *B*, the intrinsic tryptophan fluorescence of 50 μM unlabeled PNPase in the presence or absence of 20 mM MgCl_2_ was monitored with increasing concentrations of CuSO_4_. The addition of the magnesium cofactor attenuates the Cu^2+^ dose-dependent reduction in PNPase’s intrinsic tryptophan fluorescence. Error is based on two technical replicates and two biological replicates. RNase E, ribonuclease E.

At this stage, we have observed that copper associates with RNase E condensate and can lead to more solid-like assemblies. Does this change in RNase E material properties arrest or even protect client protein function? To test the impact of copper-associated condensates, we examined whether RNase E(451–898) protects PNPase activity from Cu^2+^ mismetallation. To do this, we utilized thioflavin T, which exhibits increased Stokes shift and emission intensity in the presence of poly(A) (72). Since the emission intensity scales with poly(A) concentration, it serves as a readout of PNPase activity. Without RNase E, PNPase degraded 77.5 ± 2.3% of poly(A) over 30 s when no Cu^2+^ was present. The addition of 125 μM CuSO_4_ reduced degradation to 36.4 ± 5.6%, and 250 μM CuSO_4_ further decreased it to 26.6 ± 1.2%. However, when RNase E was present, phase separation enhanced poly(A) degradation to 96.6 ± 9.4%, 94.9 ± 19.9%, and 43.3 ± 4.8% in the presence of 0 μM, 125 μM, and 250 μM CuSO_4_, respectively (Figs. 8*A* and S8*B*). These results suggest that at 125 μM, CuSO_4_, RNase E condensates preserve the activity of PNPase. Whereas higher dosages of copper lead to more solid-like assemblies that also arrest PNPase function.

Based on the PNPase activity assays, we hypothesized that RNase E condensates may provide a protective environment for client proteins to perform biochemistry in the absence of high copper stress. Indeed, copper toxicity in microbes often stems from mismetallation, impairing enzymatic functions. To investigate this, we examined copper binding to PNPase by monitoring changes in its intrinsic tryptophan fluorescence in the presence and absence of PNPase’s magnesium cofactor. With increasing concentrations of CuSO_4_, we observed a decrease in the tryptophan fluorescence signal, with apparent *K*_*d*_ values of 158.0 μM in the absence of magnesium and 301.9 μM in the presence of magnesium. However, statistical analysis did not reveal a significant difference between the line fits. This observation suggests that copper binds to PNPase in a manner that may or may not be influenced by PNPase’s essential cofactor magnesium (Figs. 8*B* and S8, *C*–*F*). In addition, we found that the presence of the histidine purification tag was not required for Cu(II) binding (S9A–D).

## Discussion

**Figure 9:**
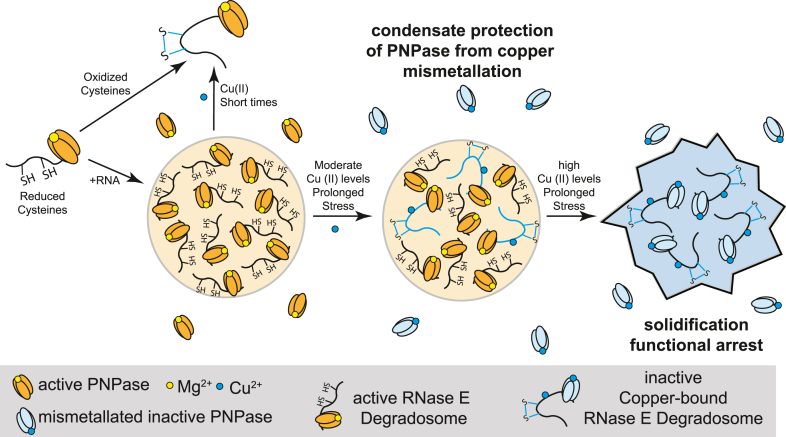
**BR-bodies act as a molecular rheostat, dynamically responding to fluctuating metal stress.** Under reducing conditions with cysteines and RNA, BR-bodies form robust, liquid-like condensates. Oxidation of cysteines by TMAD or copper drives RNase E into the dilute phase. At moderate copper levels (100–250 μM Cu^2+^), BR-bodies create protective microenvironments through direct copper binding and through enrichment of a polyphosphate that also binds copper, which prevents PNPase from copper-induced inhibition. However, at high copper concentrations (>500 μM Cu^2+^), BR-bodies initially dissolve before solidifying over time through copper-induced RNase E disulfide bond formation, arresting their function. These adaptive responses enhance *Caulobacter* fitness under copper stress. BR-body, bacterial ribonucleoprotein body; TMAD, tetramethylazodicarboxamide.

Our findings support a model in which BR-bodies function as a molecular rheostat, dynamically adjusting their functional role to protect client proteins across varying copper levels (Fig. 9). At low copper concentrations (<50 μM), RNase E condensates remain highly dynamic, supporting high cellular fitness when trace metals are depleted by chelators (Figs. 2 and S2*D*). In a moderate copper range (100–250 μM), RNase E phase separation enhances cellular fitness (Figs. 2*F* and S2*E*). *In vitro*, copper-induced condensates become more solid like (Fig. 7, *D* and *E*) while preserving PNPase activity, which would otherwise be lost because of copper exposure (Fig. 8). These findings suggest that BR-bodies create a protective microenvironment that shields PNPase and other client proteins from copper-induced inactivation, likely by providing competitive copper-binding sites that prevent mismetallation. However, at high copper concentrations (>500 μM), the condensate initially dissolves upon short exposure (Fig. 2) before transitioning into stable, solid-like assemblies *via* copper-induced disulfide bond formation (Figs. 5 and 7). Under these conditions, cell viability is severely compromised, as seen in efficiency-of-plating assays (Fig. S2*E*).

BR-bodies enhance cell fitness and preserve RNA decay activity in *Caulobacter*, potentially representing a broader design principle of how biomolecular condensates regulate metal–enzyme interactions under heavy metal stress. Indeed, a wide variety of neurodegeneration-associated condensates are also mediated by metal ions (73, 74, 75). The impact of condensates in these contexts depends on the number of metal-binding sites, their affinity, and their saturation level. When binding sites are only partially saturated and have moderate affinity, condensates may create protective zones that prevent inhibitory metal binding to client proteins. Conversely, full saturation of weak binding sites can lead to high local metal ion concentrations, which benefits some metalloenzymes while increasing toxicity and the risk of mismetallation for others.

As seen with RNase E, metal concentrations can dynamically alter condensate material properties (Fig. 7*D*), influencing the mobility and activity of client enzymes and substrates. This adaptable phase behavior allows condensates to sequester, protect, or modulate enzyme activity in response to environmental fluctuations. In addition to protecting client enzymes, such as PNPase, BR-bodies may also regulate the levels of metal-detoxifying metabolites. Our findings reveal that a subset of specialized BR-bodies colocalizes with polyphosphate granules and promotes their accumulation, either directly or indirectly, suggesting a functional link between BR-bodies and polyphosphate granules. While RNase E from *E. coli* has been observed to bind to PPK (51) and *C. crescentus* BR-bodies were found to be enriched in PPK1 (48), our observations suggest that BR-bodies and polyphosphate granules functionally coordinated during stress. This is particularly significant given polyphosphate’s established role in Cu^2+^ chelation and export, which may contribute to the enhanced copper tolerance conferred by BR-bodies. Hfq was found to heterogeneously colocalize with BR-bodies in *C. crescentus* (48), and Hfq and polyphosphate were found to form a condensate in *E. coli* that colocalizes with RNase E (26), which can bind to Hfq (76) and PPK (51). This suggests that specialized Hfq–polyphosphate BR-bodies may organize a conserved stress response across bacteria.

Future studies should investigate the crosstalk between BR-bodies and polyphosphate granules, as well as the heterogeneity or specialization of condensate, more deeply. This includes whether BR-body formation, material properties, and mRNA decay activity depend on polyphosphate, whether BR-bodies influence polyphosphate biosynthesis or stability, and how these interactions might modulate local metal ion availability to coordinate layered stress responses. Moreover, caution is warranted in biochemical studies involving condensates, metals, and the use of hexahistidine purification tags, which can inadvertently drive synthetic condensate formation (77). Collectively, understanding and applying these mechanisms could offer new insights into bacterial metal homeostasis and inform strategies for engineering stress-resistant cells in biotechnology and medicine.

## Experimental procedures

### *C. crescentus* cell growth and stress treatment

*C. crescentus* strains (*rne::rne-eyfp* or *parB::CFP-ParB; popZ::mCherry-popZ* or *rne::rne(C461A/C464C)-eyfp*) were streaked and incubated on a PYE agar plate supplemented with relevant antibiotics at 28 °C to 30 °C. When appropriate, the media were supplemented with either vanillate (5 μM), xylose (Xyl; 0.2%), gentamycin (Gent, 0.5 μg/ml), kanamycin (Kan; 5 μg/ml), chloramphenicol (2 μg/ml), spectinomycin (Spec; 25 μg/ml), and/or streptomycin (5 μg/ml).

An overnight liquid PYE culture supplemented with the appropriate antibiotics was inoculated with an individual colony for copper stress tests. The culture was grown at 220 rpm at 28 °C to the stationary phase at an absorbance of 0.9 to 1.0 at 600 nm, measured at 600 nm in a cuvette using a UV-1600PC spectrophotometer (VWR). The culture was then split into equal volumes and supplemented with 500 μM CuSO_4_, 500 μM H_2_O_2_, 500 μM TMAD, 1 mM ZnCl_2,_ and 20 mM MgCl_2_. For the CuSO_4_ titration, the culture was split into equal volumes and diluted to an absorbance of 0.2 to 0.4 at 600 nm for imaging and supplemented with various CuSO_4_ (ThermoFisher Scientific) concentrations: 0, 100, 250, 300, 350, 400, 500, 700, 800, and 1000 μM. Cell stress treatments were performed by diluting the culture to an absorbance of 0.2 to 0.4 at 600 nm for imaging before supplementation with the desired copper stress. Cells were then immediately spotted on a 1.0% PYE agarose pad. Samples were imaged at short intervals between 4 and 8 min after adding copper stress. Imaging pads were supplemented with the same concentration of CuSO_4_ as was introduced to the aliquoted liquid culture. For the CuSO_4_ washout experiments, after CuSO_4_ stress imaging, the culture was spun down for 2 min; the supernatant was removed and washed five times over 30 min with fresh PYE media. Then, the culture was repleted with fresh media and imaged to monitor potential recovery from stress. Replacement strains containing an Xyl-inducible copy of RNase E and a vanillate-inducible test construct were initially grown in media containing Xyl overnight, then washed three times with 1 ml growth media, and resuspended in growth media containing vanillate, diluted, and grown overnight. Stationary phase cultures were then diluted to an absorbance of 0.2 to 0.4 and spotted on PYE 1.5% agarose pads for imaging.

For the *Caulobacter* long-term copper exposure experiments, *C. crescentus* were diluted 1:100 from an overnight modified HIGG (mHIGG, see later) culture supplemented with appropriate antibiotics. Cells were grown to an absorbance of 0.2 to 0.3 at 600 nm at 28 °C. For strain JS49, RNase E(1–898)-eYFP is under the control of the *vanA* promoter, and cells were induced with 0.5 mM vanillate (pH 8.0) for 1 h. Cells were immobilized on a 1.0% mHIGG-agarose pad before imaging. To induce moderate copper stress at long times, strains were incubated with 1.6 mM CuSO4 for 1 h after vanillate induction at 30 °C. Cells were then immobilized on a 1.0% M2G-agarose or 1.0% mHIGG-agarose pad supplemented with 1.6 mM CuSO_4_. HIGG media were modified to generate mHIGG: 9.0 mM l-arginine (pH 7.0), 9.0 mM NH_4_Cl (pH 7.0), 8.32 mM glucose, 5 mM imidazole (pH 7.0), 1.45 mM CaCl_2_, 610.4 μM KH_2_PO_4_, 389.6 μM Na2HPO_4_, 240 mM MgCl_2_, 38.2 μM ZnSO_4_, 25.1 μM FeSO4, 10.5 μM nitrilotriacetic acid, 9.12 μM MnSO4, 6.72 μM Na_2_EDTA, 1.57 μM CuSO_4_, 852 nM Co(NO_3_)_2_, 464 nM B(OH)_3_, and 150 pM (NH_4_)_6_Mo_7_O_24_.

### *E. coli* cell growth

*E. coli* BL21(DE3) or TOP10 strains were grown at 37 °C and cultured in an LB medium (Sigma), supplemented with Kan (30 μg/ml) or ampicillin (50 μg/ml). For induction, cells were induced with IPTG (1 mM) for 2 h or 0.0004% arabinose for 1 h. Strains were analyzed at midexponential growth phase (absorbance of 0.3–0.6 at 600 nm), using a UV-1600PC spectrophotometer.

### Fluorescence microscopy and phase imaging

For cell imaging, bacterial cells were immobilized on 1.0% agarose pads made with PYE or LB media on microscope slides (3051; Thermo Scientific). Images were obtained utilizing a Nikon Eclipse Ti-E inverted microscope equipped with an Andor Ixon Ultra DU897 EMCCD camera and a Nikon CFI Plan-Apochromat 100×/1.45 oil objective and intermediate 1.5× magnification. Carl Zeiss Immersol 518 oil was used. The excitation source was a Lumencor SpectraX light engine. The filter sets utilized for YFP, CFP, and mCherry imaging were Chroma 96363, 96361, and 96322 models. YFP was excited at 512 nm, mCherry was excited at 549 nm, and emission was monitored using the CFP/YFP/mCherry Chroma filter cube. Images were acquired with Nikon NIS-Elements AR software.

For *in vitro* droplet imaging assays, 20 μM RNase E was incubated in 20 mM Tris buffer, pH 7.4 and 70 mM NaCl in the absence or presence of varying concentrations of DTT and CuSO_4_, 1 mM TMAD, and 20 mM MgCl_2_. Imaging samples were pipetted into a 1 mm well formed by an adhesive spacer (Electron Microscopy Sciences) affixed to a microscope slide (VWR) and sealed with a glass coverslip (VWR). Slides were inverted and allowed to sit at room temperature for 30 min before being placed on a Nikon Eclipse Ti-E inverted microscope with a Plan Apo-(lambda) 100×/1.45 oil objective and 518F immersion oil (Zeiss). Images were taken with an Andor Ixon Ultra 897 EMCCD camera.

### PNPase degradation assay by microscopy

Fluorescence microscopy samples were prepared by thawing requisite proteins on ice and mixing a buffer to create a final concentration of 20 mM Tris (pH 7.5), 20 mM MgCl_2_, 70 mM NaCl, 0.5 mM DTT, 5 μM purified PNPase, 25 ng/μl poly(A) RNA, and 20 μM purified wildtype RNase E(451–898) or the C461A/C464A RNase E(451–898) variant. Imaging samples were pipetted into a 1 mm well formed by an adhesive spacer (Electron Microscopy Sciences) affixed to a microscope slide (VWR) and sealed with a glass coverslip (VWR). Slides were inverted, and samples lacking PNPase were allowed to sit at room temperature for 30 min before imaging. Samples with PNPase and poly(A) were also imaged within 30 min of plating on a Nikon Eclipse Ti-E inverted microscope with a Plan Apo-(lambda) 100×/1.45 oil objective and 518F immersion oil (Zeiss). Images were taken with an Andor Ixon Ultra 897 EMCCD camera.

### Fluorescence image processing

Cell image analysis was conducted using the MicrobeJ software (78). A set of images of each unique condition was uploaded to MicrobeJ, consisting of a combination of phase contrast, YFP, CFP, and mCherry channels when appropriate. The phase image was utilized to create a cell outline using the Medial Axis setting. For automated foci detection, the maxima foci function of MicrobeJ was used, and the tolerance setting was manually adjusted between 250 and 1000 to identify and outline foci on a test image. The Z-score, a parameter indicative of the number of standard deviations from the mean, was set at 20.0. The segmentation tool was then utilized to split adjoined foci, and aberrant foci with an area <0.01 μm^2^ and length >1 μm were omitted. A Welch’s two-tailed *t* test using unequal variances was subsequently performed on all unique samples compared with the control. For each set of images uploaded, the same tolerance/Z-score parameters were used to quantify the average foci/cell. To determine partition ratios, we background-subtracted the RNase E maximum (foci) and minimum intensities within the cell bodies as calculated by MicrobeJ. We divided the maximum intensity of the YFP signal in all foci by the YFP intensity within the cell apart from the foci.

Channel 1 was designated as “Bright,” and the following parameters were selected: “Exclude on Edges,” “Shape descriptors,” “Segmentation,” “Edge Correction,” “Intensity,” and “Shape.” In addition, the area was bound by 0.2-max μm^2^, length 1-max, width 0-max, circularity 0 to 1, and curvature, sinuosity, angularity, solidity, and intensity 0-max. Channel 2 was designated as “Dark,” and the following parameters were selected: area was bound by 0-max μm^2^, length 0 to 2.81, width 0-max, circularity 0 to 0.74, and intensity 1710-max.

### mRNA half-life measurements

*C. crescentus* NA1000 cells were grown in liquid PYE at 28 °C to an absorbance of ∼0.3 to 0.6 at 600 nm (exponential phase). Copper II sulfate was added to the cultures at the final concentration of 0 mM, 125 mM, or 500 mM for 8 min before the RNA extraction.

Regarding *JS38* and *JS802*, cells were first grown overnight in liquid PYE + Kan + Gent + 0.2% Xyl at 28 °C. Once the absorbance reached ∼0.3 to 0.6, cells were washed three times with PYE and resuspended in 25 ml PYE at an absorbance of 0.05. Vanillate (500 μM) was added, and the cultures were incubated for 8 h before the RNA extraction.

Cells (1 ml) were taken at time 0 (before the addition of rifampicin) and vortexed with a milliliter of RNAprotect Bacterial reagent. Following the addition of rifampicin (200 μg/ml), 1 ml aliquots were collected at predetermined intervals (1, 2, 4, and 8 min) and vortexed with 2 ml of RNAprotect Bacterial reagent as previously. Cells were then pelleted by centrifugation at 6000 rpm for 2 min and resuspended in 1 ml 65 °C prewarmed TRIzol. The samples were then incubated at 65 °C for 10 min. Afterward, 200 μl of chloroform was added to the samples, and the tubes were inverted six to seven times. The samples were incubated for 5 min at room temperature before being centrifuged for 10 min at maximum speed. The temperature of TRIzol was set at 65 °C. After centrifuging the cells in RNAprotect for 2 min at 8000 rpm, they were resuspended in 1 ml of TRIzol that had been warmed up beforehand and incubated for 10 min at 65 °C. The upper aqueous phase was then pipetted into a new tube. Two microliters of 1 mg/ml glycogen and 700 μl of isopropanol were added to the samples and incubated at −80 °C overnight. The following day, the samples were centrifuged for 1 h at maximum speed at 4 °C. The supernatant was discarded, and the RNA pellets were washed with 1 ml of ice-cold 80% ethanol. The samples were then centrifuged for 10 min at maximum speed. The ethanol was discarded, and the RNA pellets were air-dried and resuspended in 50 μl of RNA resuspension buffer (10 mM Tris–HCl, pH 7.0, 0.1 mM EDTA).

For measuring mRNA half-lives by RT–quantitative PCR, a master mix was prepared, including 1× Luna Universal One-Step Reaction Mix, 1× Luna WarmStart RT Enzyme Mix, 0.4 μM of forward and reverse primers, and Milli-Q water. RNA (100 ng/μl) from each collected sample was aliquoted into a 96-well plate and mixed with 19 μl of the master mix. The mixture was briefly vortexed and centrifuged. A QuantStudio Real-Time PCR machine was used to conduct the RT–quantitative PCRs. RNA decay rates were determined by fitting a linear curve to the natural logarithm of the proportion of RNA that remained at each time point. Each time point's RNA quantity was standardized to the 100% amount found in the time 0 samples. The mRNA half-life was calculated using linear regression of the ln (% of RNA remaining) at each RNA extraction time point. The slope was then converted to a half-life measurement using the formula t1/2 = -ln (2)/slope.

### Ellman assay

Reaction buffer (100 mM Tris–Cl, pH 8.0) was prepared and used to generate stocks of DTT (1.5 mM) and 5,5′-dithiobis(2-nitrobenzoic acid) (DTNB, 5 mM in 50 v.% dimethyl sulfoxide). All reagents were stored on ice before use. A set of DTT standards were created covering 0 to 100 μM DTT dissolved in reaction buffer before 200 μM DTNB was added and allowed to incubate at room temperature for 15 min. The absorbance at 410 nm was measured on a NanoDrop 2000C, and a line of best fit was generated: absorbance at 410 nm = 0.001468·[SH] + 0.0007433 (R2 = 0.9984), where [SH] represents the concentration of free sulfhydryl groups in micromolar. Error bars represent the results of two replicates.

For each condition, 5 to 10 μM RNase E(451–898), RNase E(451–898, C461A/C464A) (AEHA mutant), or RNase E(1–898, D403C) (FL-RNE) was incubated with 200 μM DTNB for 15 min in 100 mM Tris–Cl, pH 8.0, before an absorbance at 410 nm measurement. Protein was mixed with either a fivefold excess of Tris(2-carboxyethyl)phosphine for 30 min (reduced; AEHA mutant), a 20-fold excess of TMAD for 1 h (oxidized), a 10-fold excess of metal for 2 min, or phase separation was induced with either 10 wt% PEG (8000) or 20 mM MgCl_2_ before DTNB addition. Significance was determined using a one-way ANOVA compared with the results of RNase E(451–898).

Metal stocks were diluted immediately before use in 100 mM Tris–Cl, pH 8.0, except for CuCl, which was diluted into 7.5 M NH_4_OAc. Metal sources included AlCl_3_, CaCl_2_, CoCl_2_, CuCl, CuSO_4_, FeSO_4_, FeCl_3_, MgCl_2_, MnCl_2_, NiSO_4_, and ZnSO_4._ The concentration of sulfhydryl groups was divided by the concentration of RNase E to obtain the number of sulfhydryl groups per protein. An error was determined based on two replicates.

### BCS assay for Cu^1+^

Reaction buffer (100 mM Tris–Cl, pH 8.0, 1 mM DTT) was prepared and used to dilute the 10 mM BCS. CuCl stocks (100 μM–100 mM) were generated by dilution into 7.5 M NH_4_OAc. All reagents were stored on ice before use. A set of Cu^1+^ standards was created in triplicate, covering 0 to 100 μM Cu^1+^ dissolved in reaction buffer, before 500 μM DTNB was added and allowed to incubate at room temperature for 2 min. The absorbance at 483 nm was measured on a NanoDrop 2000C, and a line of best fit was generated: absorbance at 483 nm = 0.001326·[Cu^1+^] + 0.0009528 (*R*^2^ = 0.9983), where [Cu^1+^] represents the concentration of free Cu^1+^ in micromolar. Error bars represent the results of two replicates. To measure the Cu^1+^ content after incubation of Cu^2+^ with RNase E in solution, the protein was treated with a fivefold excess of Tris(2-carboxyethyl)phosphine before being desalted into 200 mM NaCl, 100 mM Tris–Cl, pH 8.0. The protein was then incubated with a 10-fold excess of CuSO_4_ in 200 mM NaCl, 100 mM Tris–Cl, pH 8.0 for 2 min before adding BCS. For each 10 μM RNase E sample, 500 μM BCS was added, and an absorbance at 483 nm measurement was immediately taken. An error was determined based on two replicates.

### Thioflavin T assay for tracking poly(A) degradation

For reactions without RNase E, samples were prepared in the following order, with final concentrations as listed: 20 mM Tris–Cl (pH 7.5), 70 mM NaCl, 2.5 mM PNPase, 500 μM MgCl_2_, 25 ng/μl poly(A), 10% PEG-8000, and 4 mM Na_2_HPO_4_ (phosphate). Phosphate was added last to initiate the reaction, which proceeded for 30 s before quenching. The reaction was quenched with final concentrations of 100 mM EDTA, 25 μM thioflavin T, and 556 mM NaCl.

RNase E was added before PNPase to a final concentration of 20 μM for reactions with phase separation. In reactions with copper, CuSO_4_ was introduced after PEG-8000, reaching final concentrations of either 125 μM or 250 μM.

To generate a standard curve for each condition, MgCl_2_ was omitted, and intensity measurements were taken with the following poly(A) concentrations: 20 ng/μl, 10 ng/μl, 5 ng/μl, and 0 ng/μl.

Fluorescence measurements were conducted using a Tecan Infinite M1000 microplate reader (Tecan Group Ltd). Samples were prepared in a 96-well U-bottom microplate (Greiner Bio-One). Fluorescence detection was performed in top-read mode with an excitation wavelength of 438 nm and an emission wavelength of 491 nm, both with a bandwidth of 5 nm. The instrument was set to manual gain mode with a fixed gain of 135. Each well was measured using 50 flashes at 400 Hz, and the Z-position was set to 18,400 μm to optimize signal detection.

To calculate the percentage of poly(A) degraded, 10 intensity measurements were taken for three replicates of each condition after the reactions were quenched. The mean intensities for each replicate were associated with the poly(A) concentrations according to the standard curve fits of each condition. The standard deviations of each intensity measurement were calculated by propagating the uncertainties through a first-order Taylor expansion method given by:

$$\sigma_{f}=\frac{\partial f\left(I\right)}{\partial I}\sigma_{I}$$

Here, f(I) is the concentration of poly(A) based upon the standard curve fit; I is the measured mean intensity from a particular replicate; and $\sigma_{I}$ is the standard deviation of I. Finally, given a starting poly(A) concentration of 25 ng/μl:

$$\%\;degraded=\left(\frac{25\frac{ng}{\mu L}-fit\;value}{25\frac{ng}{\mu L}}\right)\times 100\%$$

$$\sigma_{f}\left(\%\right)=\frac{\sigma_{f}}{25\frac{ng}{\mu L}}\times 100\%$$

### Fluorescence recovery after photobleaching

FRAP experiments were conducted using a Nikon Ti2 microscope equipped with a Yokogawa CSU spinning disk confocal system and an Andor DU-897 EMCCD camera. A Plan Apo λ 100× oil-immersion objective (numerical aperture = 1.45) was used to acquire images with a pixel resolution of 512 × 512. The sample was illuminated using an LU-NV NIDAQ multi-laser system, with the 515 nm excitation laser line set at 40% power. The emission was collected through a 593.5 nm emission filter and a dichroic mirror (86,006—CFP/YFP/DsRed).

Images were captured at a 70 ms exposure time. The EMCCD camera was set to a gain multiplier of 80, a readout speed of 17 MHz, and a vertical shift speed of 3.3 μs with a vertical clock voltage amplitude of +4V. For FRAP, a region of interest within the condensates was photobleached using a Galvo XY scanning system. Fluorescence recovery was monitored over 63 time points. The time interval between acquisitions was 10 s, and the acquisition rate was approximately 47.5 frames per second. Data analysis was performed by normalizing fluorescence intensities to prebleach values and fitting the recovery kinetics using a single exponential model.

### PNPase-mediated RNA-degradation assay

RNA-degradation assays were performed at room temperature in 20 mM Tris–HCl (pH 7.5), 70 mM NaCl, 20 mM MgCl_2_, 4 mM Na_2_HPO_4_ (pH 7.5), and 0.5 mM DTT with 5 μM purified PNPase and 20 μM purified unlabeled wildtype RNase E CTD or the unlabeled C461A/C464A RNase E variant, in addition to 100 μM, 250 μM, or 500 μM CuSO_4,_ when appropriate. Reactions were initiated by adding 5 μM PNPase to a mixture containing 25 ng/μl poly(A) RNA. For time-course assays, aliquots were withdrawn and quenched in 100 mM EDTA. Samples were denatured in 1.5 volumes of 2× RNA loading buffer containing 95% formamide, 18 mM EDTA, and 0.025% SDS and incubated at 95 °C for 3 min. Quenched ribonuclease reaction aliquots were loaded onto a prerun 6% acrylamide gel containing 7 M urea in 1× TBE buffer (89 mM Tris base, 89 mM boric acid, and 2 mM EDTA). To separate RNA, the gel was run in 1× TBE at 250 V at room temperature. Subsequently, the gel was rinsed in Milli-Q water for 5 min and stained for 20 min with 1× SYBR Gold nucleic acid stain (Invitrogen) in 1× TBE. Each gel assay included RNA-only and protein-only controls. Gels were imaged using the Bio-Rad ChemiDoc MP imager with SYBR Gold settings and quantified using the Bio-Rad ImageLab software. The intensity of the protein-only lane was subtracted from each timepoint lane intensity and converted to nanograms of poly(A) in correlation with intensity based on the intensity in the lane with the starting amount of poly(A) and plotted against time (seconds). The degradation rate was calculated using the initial time points up to 60 s for which a line of best fit was generated. From this equation, the slope was obtained to correlate to a degradation rate of poly(A) (ng/s). This rate was then converted to ng RNA/min/mg PNPase by multiplying this rate by 60 s/min and dividing it by the amount of protein in milligramss.

### Protein expression and purification of PNPase

Protein expression and purification were completed as described previously (4). Briefly, plasmid pMJC0094 was constructed to heterologously express an N-terminally His_6_-tagged *C. crescentus* PNPase in *E. coli*. Plasmid pMJC0094 was transformed into chemically competent Rosetta (DE3) cells, plated onto LB–Miller plates supplemented with 50 μg/ml ampicillin, and incubated overnight at 37 °C. An overnight 60 ml LB–Miller culture was inoculated from a single colony incubated at 37 °C. From this saturated culture, 6 l of LB–Miller media was inoculated with 60 ml of the saturated culture and grown at 37 °C to midlog phase (absorbance ∼0.5 at 600 nm). The expression of PNPase was induced with 333 μM IPTG for 4 h at 25 °C. The cells were collected by centrifugation at 4 °C, 4000*g*, for 30 min. The resulting pellet was washed with 60 ml resuspension buffer (50 mM Tris [pH 7.5], 500 mM NaCl) before being pelleted at 4 °C, 4000*g*, for 20 min and stored at −80 °C. The cell pellet was thawed on ice and then resuspended in 10 ml lysis buffer per liter of culture (20 mM Tris–HCl [pH 7.5], 500 mM NaCl, 5 mM imidazole, and 200 U benzonase) supplemented with SigmaFast protease inhibitor tablets (Sigma). The cell suspension was lysed by continuous passage through an Avestin Emulsiflex-C3 at 15,000 psi for 15 min at 4 °C. Cell debris was pelleted by centrifugation at 20,000*g* for 45 min at 4 °C. The supernatant was loaded onto a HisTrap FF column (GE Healthcare) and washed with 20 column volumes of wash buffer (20 mM Tris–HCl [pH 7.5], 500 mM NaCl, and 5 mM imidazole). Then, it was eluted with elution buffer (20 mM Tris–HCl [pH 7.5], 500 mM NaCl, and 500 mM imidazole). The His_6_-tag was cleaved off with tobacco etch virus (TEV) protease. Fractions containing PNPase were supplemented with 50 mM sodium phosphate (pH 7.5) at 37 °C for 1 h to drive phosphorolysis of copurifying RNA. The fractions were loaded onto a G-Sep 6 to 600 kDa Size-Exclusion Columns (G-Biosciences) and eluted with storage buffer (20 mM Tris–HCl [pH 7.5], 200 mM NaCl, and 5% [v/v] glycerol). Fractions containing PNPase were concentrated using 50,000 molecular weight (MW) cutoff Amicon centrifugal filters to 15.5 mg/ml (MW: 79,530 g/mol, ε: 42,985 1/M·cm), aliquoted, and flash-frozen in liquid nitrogen and stored at −80 °C.

### Purification of RNase E-CTD

*C. crescentus* RNase E(451–898) was expressed in *E. coli* Rosetta BL21(DE3) from a plasmid (pET28::His6-MBP-TEV-RNase E(451–898)/WSC1654) possessing a His_6_-tag and MBP fusion. Protein expression was induced in cells grown to midlog phase (absorbance at 600 nm ∼0.5) at 37 °C with 1 mM IPTG for 4 h before harvesting. Cells were frozen at −80 °C prior to purification after washing in 500 mM NaCl, 50 mM Hepes–KOH, pH 8.0, and repelleting. Harvested frozen cells were thawed on ice and then resuspended in 10 ml lysis buffer per liter of culture (20 mM Tris–HCl [pH 7.5], 500 mM NaCl, 5 mM imidazole, 1 mM β-mercaptoethanol, 5% glycerol, 100 U DNase I, and 0.1% Triton X-100) supplemented with SigmaFast protease inhibitor tablets (Sigma). The cell suspension was lysed by continuous passage through an Avestin Emulsiflex-C3 at 15,000 psi for 45 min at 4 °C. Cell debris was pelleted by centrifugation at 20,000*g* for 45 min at 4 °C. The supernatant was loaded onto a HisTrap FF column (GE Healthcare) and washed with 20 column volumes of wash buffer (5 mM Tris–HCl [pH 7.5], 500 mM NaCl, 5 mM imidazole, 1 mM β-mercaptoethanol, and 5% glycerin). Subsequently, the purification was eluted with elution buffer (20 mM Tris–HCl [pH 7.5], 200 mM NaCl, 200 mM imidazole, 1 mM beta-mercaptoethanol, and 5% glycerin). The His_6_-tag and MBP-tag were cleaved off with TEV protease before being loaded onto a 16/60 Gel Filtration column to separate MBP from RNase E. Fractions containing RNase E were loaded onto a G-Sep 6 to 600 kDa Size-Exclusion Columns and eluted with storage buffer (20 mM Tris–HCl, pH 7.5, and 200 mM NaCl). RNase E's fractions were concentrated using 30,000 MW cutoff Amicon centrifugal filters (MW: 49,659 g/mol, ε: 32,095 1/M·cm), aliquoted, and stored at −80 °C.

### RNase E-CTD EPR sample preparation

Purified RNase E protein was thawed and buffer exchanged by a 2 h followed by an overnight dialysis in Hepes buffer (20 mM Hepes [pH 7.5], 200 mM NaCl). The protein stock was then concentrated to 267.04 μM. CuSO_4_ was added and allowed to incubate at room temperature for 30 min. CW-EPR samples for analysis were then prepared to a total volume of 100 μl with 20 v% glycerol at a final RNase E concentration of 120 μM. Samples were then flash-frozen in liquid MAPP gas and stored at −80 °C.

### EPR experiments

#### Continuous wave electron paramagnetic resonance

CW-EPR experiments were performed at 80 K with a Bruker E580 X-Band FT/CW spectrometer, a Bruker ER4118X-MD5 resonator, Oxford CF935 dynamic continuous-flow cryostat, and Oxford LLT 650 low loss transfer tube. The spectra were collected at a microwave frequency of ∼9.67 GHz with a 30 dB attenuation, 4 G modulation amplitude, 100 kHz modulation frequency, and 20.48 ms conversion time. The magnetic field sweep was centered at 3100 G and was 2000 G long, collected with 1024 data points and 200 scans. All simulations were performed using the pepper function in EasySpin Software.

#### Electron spin-echo envelope modulation EPR

Three-pulsed ESEEM experiments were performed at 18 K with a Bruker E580 X-Band FT/CW spectrometer, a Bruker ER4118X-MD4 resonator, Oxford CF935 dynamic continuous-flow cryostat, and Oxford LLT 650 low loss transfer tube. A pulse sequence of π/2 – τ - π/2 – T - π/2 – echo with a π/2 pulse width of 10 ns was used, where t = 140 ns, T = 280 ns, and was incremented in steps of 16 ns. The ESEEM experiments were carried out at 3340 G and 3480 G. The raw experimental data were background-subtracted using a stretched exponential decay, and the Fourier transformed time domain signal was calculated using the Bruker XEPR software.

### Tryptophan fluorescence quenching analysis

Tryptophan fluorescence data were obtained using a FluoroMax-3 fluorimeter (Jobin Yvon Horiba). Samples with unlabeled wildtype or mutant C461A/C464A RNase E(451–898) with or without PNPase were prepared by incubating 50 μM RNase E and 50 μM PNPase in 100 mM NaCl, 20 mM Tris, pH 7.4 with varying amounts of CuSO_4_ in a total volume of 70 μl. Measurements were taken using a quartz cuvette. The FluorEssence software was used to collect the data (Jobin Yvon Horiba), with an excitation wavelength set to 280 nm and emission wavelengths scanned between 300 and 450 nm. Maximum fluorescence intensities at 359, 358, and 350 nm were used to estimate the apparent *K*_*D*_ of Cu^2+^ interactions with RNase E(451–898), AEHA Mutant RNase E(451–898), and PNPase, respectively.

### EOP assays

All *C. crescentus* strains used in this study were derived from the wildtype strain NA1000 (ΔCTD and ΔDBS as the only copy of RNase E on the chromosome, which do not pick up suppressor mutations) and were grown at 28 °C in PYE medium (79). When appropriate, media were supplemented with vanillate (5 μM), Xyl (0.2%), Gent (0.5 μg/ml), Kan (5 μg/ml), chloramphenicol (2 μg/ml), Spec (25 μg/ml), and/or streptomycin (5 μg/ml). Strains were analyzed at stationary (absorbance = 0.9–1.2) and at exponential growth phase (absorbance = 0.1–0.3). Absorbance was measured at 600 nm in a cuvette using a UV-1600PC spectrophotometer. For the EOP assays, cells were grown overnight in PYE, which lacked antibiotics and was diluted to an absorbance of 0.05 at 600 nm. Serial dilutions (10-fold) were then performed and spotted on plates with a specified concentration of ethanol or CuSO_4_. After spotting cells, the plates were allowed to sit with the agar side down for 30 min to dry before the plates were incubated, inverted, at 28 °C for 1 to 3 days.

All *A. tumefaciens* strains used in this study (JS5 (3) and JS376) were derived from the wildtype strain C58 and were grown at 28 °C in LB medium supplemented with Gent (30 μg/ml). Strains were analyzed at exponential growth phase (absorbance= 0.3). For the EOP assays, cells were grown overnight in LB + gent and were diluted to an absorbance of 0.05 at 600 nm. Serial dilutions (10-fold) were then performed and spotted on LB plates either lacking CuSO_4_ or containing 3 mM CuSO_4_. After spotting cells, the plates were allowed to sit with the agar side down for 30 min to dry before the plates were incubated, inverted, at 28 °C for 1 to 3 days.

### *In vivo* DAPI staining of BR-bodies

To stain with DAPI, cells (WSC 1358, rne::rne-eyfp) were grown overnight to an absorbance of 0.8 and treated with 12 μg/ml DAPI, added directly to the culture medium, and incubated at 30 °C in the dark in a rotator for 25 min as described previously (54). Cells exposed to copper stress (100 μM and 250 μM) were incubated with copper stress for short (10 min) or long (2 h) exposure prior to the addition and incubation of DAPI for 25 min. Cells were then diluted to an absorbance of 0.2 to 0.4 prior to being spotted on 1% agarose pads for microscopy. Fluorescence microscopy was conducted with a Nikon Eclipse Ti-E inverted microscope with a Plan Apo-(lambda) 100×/1.45 oil objective and 518F immersion oil (Zeiss). Images were taken with an Andor Ixon Ultra 897 EMCCD camera. A custom filter set was used to visualize DAPI-polyP, using 390/70-nm excitation filter, a 488-nm dichroic, and a 515-nm long-pass emission filter.

### PAR assay for detection of metal binding *via* free thiols

All solutions were prepared using Milli-Q ultrapure water. Metal stock solutions (100 mM) of Cu^2+^ and Zn^2+^ were prepared by dissolving the appropriate amount of each metal in water using a volumetric flask. Working metal stock solutions (2 mM) were prepared volumetrically by further dilution with water. A stock solution of PAR (1.6 mM) was prepared by dissolving the chelator in water with Hepes (C_8_H_18_N_2_O_4_S) (222 mM). The pH of the PAR solution was adjusted to 8.0 using NaOH pellets. The PAR stock solution was stored in plastic conical vials covered in aluminum foil at 4 °C to minimize compound degradation. For metal determination, PAR from the PAR stock solution at a final concentration of 800 μM was mixed with protein at a final concentration of 100 μM and incubated in the presence and absence of 50 μM of metal (Cu^2+^ or Zn^2+^) for 30 min. For protein metal determination, absorbance scans were subsequently measured from 300 nm to 600 nm at room temperature on a plate reader, and absorbance at 500 nm was plotted to correlate to free metal concentration based on a standard curve constructed for each metal of absorbance at 500 nm *versus* metal concentration. Once absorbance values were correlated to free metal concentration, bound metal concentration was determined by subtracting total final concentration of metal initially added from the free metal concentration determined from the standard curve. Fluorescence measurements were conducted using a Tecan Infinite M1000 microplate reader (Tecan Group Ltd). Samples were prepared in a 96-well U-bottom microplate (Greiner Bio-One).

### Statistical analysis

All data are presented as mean ± SD, with individual data points shown where applicable. For comparisons between two independent groups, an unpaired two-tailed Student’s *t* test was performed (Figs. 3*B*, 4*F* and 5*B*). For comparisons involving more than two independent groups with a single factor, an ordinary one-way ANOVA was conducted to assess overall differences among means. When the ANOVA *F*-test was significant, pairwise group comparisons were performed using Tukey’s honestly significant difference *post hoc* test (Figs. 3*D*, 5*C*, 8*A*, S1*H*, S2, *B*, *C*, S3*B* and S4*B*).

For both *t* tests and Tukey’s *post hoc* comparisons, statistical significance was defined as *p* ≤ 0.05 (∗), *p* ≤ 0.01 (∗∗), and *p* ≤ 0.001 (∗∗∗). *p* > 0.05 was considered not significant (ns). All experiments were performed with at least three independent biological replicates, which were averaged prior to statistical analysis. Data analysis and visualization were performed using GraphPad Prism (version 10.2.0; GraphPad Software).

### JS801 rne::rneΔDBS SpecR

The CTD region lacking the DBSs of the gene specifying RNase E (RNE) was amplified using primer HY17F ggtcgatgacagcctgcacgcgggcgac and HY17R aaagatcttacggcgcggtgatctcgttcg from the pv*RNE(ΔDBS)-YFP* GentR. The HY16F caccgcgccgtaagatcttttctacggggtctg and HY16R cgtgcaggctgtcatcgaccacgaccgac primers were used to amplify a pNTPs vector containing 1 KB regions upstream and downstream of RNE’s NTD and a Spec cassette from the RNEΔCTD SpecR pNTPS138 plasmid (80). After running the DNA fragments on a 1% agarose gel, a GeneJet Gel Extraction kit was used to extract the PCR products. The GeneJET PCR Purification Kit was then used to purify the vector sample after it had been treated with Dpn1. The CTDΔDBS insert region was then assembled into the RNEΔCTD SpecR pNTPS138 vector *via* Gibson Assembly (NEB). The Gibson mix was then used to transform chemically competent *E. coli* DH10B cells that were then selected on LB agar plates supplemented with Kan (30 ug/ml). Kan-resistant colonies were screened for the presence of the CTDΔDBS insert region by PCR using the HY18F taagatcttttctacggggtctg and HY18R tcttcttcctcgtcgtcg primers and verified by Sanger sequencing (Genewiz).

For the RNEΔDBS strain, the purified RNEΔDBS SpecR pNTPS138 (pHY001) plasmid was mated into *JS769* cells (80) *via* triparental mating and plated on PYE agar plates supplemented with nalidixic acid + Spec + Kan (100 μg/ml Spec; 20 μg/ml nalidixic acid; and 25 μg/ml Kan). The resulting kanR/specR resistant colonies were then inoculated into plain PYE and then counter-selected on PYE plates supplemented with Spec + 3% sucrose. Colonies that were resistant to Spec and sensitive to Kan were then identified and screened by PCR using primers Screen F tcggtcggcctctatatcc and Screen R gcgaaccctgaccaatctaa for the deletion of the DBS regions.

### JS495 VanA:: (double cys mutant(CTD)-YFPC-4) GentR

The RNE DNA fragment with the double cysteine-to-alanine mutations was synthesized (CATATGTCGAAGAAGATGCTGATCGACGCAGCACACGCCGAAGAGACGCGTGTGGTCGTCGTGGACGGTACCCGGGTTGAAGAGTTCGATTTCGAGAGCCAAACCCGCAAACAGCTTCGTGGAAACATCTATCTCGCCAAGGTGACGCGCGTTGAGCCCAGCCTCCAGGCTGCGTTCATCGAGTACGGCGGCAACCGTCACGGTTTCCTGGCGTTCAACGAGATCCACCCCGACTACTACCAGATCCCGGTCGCCGACCGCGAAGCGCTGATGCGCGACGACTCCGGTGACGACGAGGACGACACCCCGATCTCGCGTCGCGCCTCCGGCGGCGACGACGAAGACGACGTCAATGGCGGCGACCGCGCGGTCGACGATGATGACGATGACGTCGAAGAAGAACTGGCGCGCCGCAAGCGCCGCCTGATGCGCAAGTACAAGATCCAGGAAGTGATCCGCCGCCGGCAGATCATGCTGGTTCAGGTGGTCAAGGAAGAGCGTGGCAACAAGGGCGCGGCCCTGACCACCTATCTGTCGCTGGCCGGCCGCTACGGCGTCCTGATGCCCAACACCGCCCGTGGCGGCGGCATCAGCCGCAAGATCACGGCGGTGACTGACCGCAAGCGCCTGAAGAGCGTCGTCCAAAGCCTGGACGTGCCGCAAGGCATGGGTCTGATTGTCCGCACAGCCGGCGCCAAGCGCACCAAGGCCGAGATCAAGCGCGACTATGAGTACCTGCTGCGTCTGTGGGAGAACATCCGCGAGAACACGCTGCACTCGATCGCGCCGGCGCTGATCTACGAGGAAGAAGACCTCGTCAAACGCGCCATCCGCGACATGTACGACAAGGACCTGGACGGCATCTGGGTCGAGGGCGACGCCGGCTACAAGGAAGCGCGCGACTTCATGCGCATGCTAATGCCGAGCCAGGCCAAGAAGGTCTTCAACTACCGCGACCCGACCCCGCTGTTCGTGAAGAACAAGATCGAGGACCATCTGGCCCAGATCTATTCGCCGGTCGTTCCGCTGCGCTCGGGCGGCTATCTGGTGATCAACCAGACCGAGGCCCTGGTCGCCATCGACGTCAACTCGGGTAAGGCCACGCGCGAGCGCAACATCGAGGCCACCGCGCTGAAGACCAACTGCGAAGCGGCCGAGGAAGCCGCCCGTCAGCTGCGTCTGCGCGACCTGGCCGGCCTGATCGTCATCGACTTCATCGACATGGATGAAGCCAAGAACAACCGCACGGTCGAGAAGGTCCTGAAGGACGCGCTCAAGGACGACCGCGCGCGCATCCAGATGGGCAAGATCTCGGGCTTTGGCCTGATGGAGATCAGCCGTCAGCGTCGCCGCACCGGCGTGCTGGAAGGCACCACCCATGTCGCCGAACACGCCGAAGGCACCGGCCGTGTCCGTTCGGTGGAATCCAGCGCCCTGGCCGCCCTGCGCGCCGTCGAGGCCGAGGCCCTGAAGGGCTCGGGCAGCGTGATCCTGAAGGTCTCGCGCTCGGTCGGCCTCTATATCCTCAACGAAAAGCGCGATTATCTGCAGCGTCTGCTGACCACGCACGGCCTGTTCGTGTCGGTCGTGGTCGATGACAGCCTGCACGCGGGCGACCAGGAGATCGAGCGCACCGAGCTGGGCGAACGCATCGCCGTGGCCCCGCCGCCCTTCGTCGAGGAAGACGACGACTTCGATCCGAACGCCTACGACGACGAGGAAGAAGAAGACGACGTCATTCTCGATGACGAGGACGACACCGACCGCGAGGACACCGACGACGACGATGCGACGACGCGCAAGTCGGCGCGTGATGACGAGCGCGGCGACCGCAAGGGCCGTCGCGGGCGTCGCGACCGCAACCGCGGCCGCGGGCGTCGCGACGAGCGGGATGGCGAGACCGAGTCCGAGGACGAGGACGTCGTGGCCGAAGGCGCGGACGAGGATCGCGGCGAGTTTGGCGATGATGATGAAGGCGGTCGTCGCCGCCGTCGCCGGGGTCGTCGTGGCGGCCGTCGTGGCGGGCGCGAGGACGGCGATCGTCCGACCGACGCCTTCGTCTGGATCCGTCCGCGGGTGCCCTTCGGCGAGAACGTCTTCACCTGGCATGATCCGGCTGCGCTGGTCGGCGGTGGCGAGTCGCGTCGTCAGGCGCCCGAGCCGCGCGTCGATGCCGCTACCGAGGCCGCGCCGCGTCCCGAGCGGGCCGAGCGCGAAGAGCGCCCTGGCCGTGAACGTGGCCGTCGGGGTCGTGACCGGGGCCGTCGCCAGCGCGACGAGGCGCCGGTCGCCGAGATGACCTCGGTGGAAAGCGCGACTGTCGAGGCTGCGGAGCCGTTCGAGGCCCCCATCCTGGCGCCGCCGGTAATCGCCGGGCCGCCGGCCGACGTTTGGGTCGAACTGCCGGAAGTCGAGGAAGCGCCCAAGAAGCCCAAGCGCTCAAGGGCGCGCGGCAAGAAAGCGACTGAAACGTCCGTCGAAGCGATCGACACCGTCACCGAAGTCGCGGCGGAGGCTCCCGCCCCCGAGACCGCTGAACCCGAAGCCGTCGAGGTCGCTCCGCCGGCCCCCACGGTCGAGGCTGCGCCTGAGCCGGGACCGGTCGTCGAAGCCGTCGAGGAGGCCCAACCGGCCGAGCCGGATCCGAACGAGATCACCGCGCCGCCCGAAAAGCCCCGTCGGGGCTGGTGGCGCCGGGCGAATTC) and subcloned into pVYFPC-4 by GenScript. The resulting RNE(Cys-to-ala)-pVYFPC-4 plasmid was transformed into NA1000 cells and selected on PYE plates supplemented with Gent (5 μg/ml). The resulting GentR colonies were grown in liquid PYE + Gent (0.5 μg/ml).

### JS802 rne::pXRNEssrAC VanA:: (double cys mutant(CTD)-YFPC-4) KanR GentR

Phage lysate from strain *JS8* harboring RNE KanR under the Xyl promoter was transduced into JS495 cells and selected on PYE + Gent + Kan + Xyl plates. The resulting GentR KanR colonies were inoculated into liquid PYE supplemented with Gent + Kan + Xyl.

### *JS376* rne::rne*ΔCTD*-eYFP gentR

The *Agrobacterium* RNase E gene (525 bp) up to the codon 692 was PCR amplified from the *A. tumefaciens* genome using primers agro_ntd-R gcgtaacgttcgaattcgcatcgtcttcttcttcaacgaagctcgg and agro_ntd-F cgtccaattgcatatgcttgctggtctcgttgtcatcg. The PCR product was digested with NdeI and EcoRI and ligated into pYFPC-4 (81) and verified by Sanger sequencing. The resulting plasmid was then electroporated into *A. tumefaciens* as in the study by Al-Husini *et al.* (3).

## Data availability

We declare that the data supporting the findings of the study are available within this article and its Supporting Information Files or from the corresponding author (W. S. C.). In addition, the plasmids and strains used in this study are available from the corresponding author (W. S. C.) upon request.

## Supporting information

This article contains supporting information.

## Conflict of interest

The authors declare that they have no conflicts of interest with the contents of this article.
