# Supplementary material for: BR-bodies facilitate adaptive responses and survival during copper stress in Caulobacter crescentus[image]
Source: J Biol Chem. 2025 Aug 28;301(10):110648. doi: 10.1016/j.jbc.2025.110648 (PMC12510024; doi:10.1016/j.jbc.2025.110648)
Supplement: Supplementary Data [file mmc1.docx]

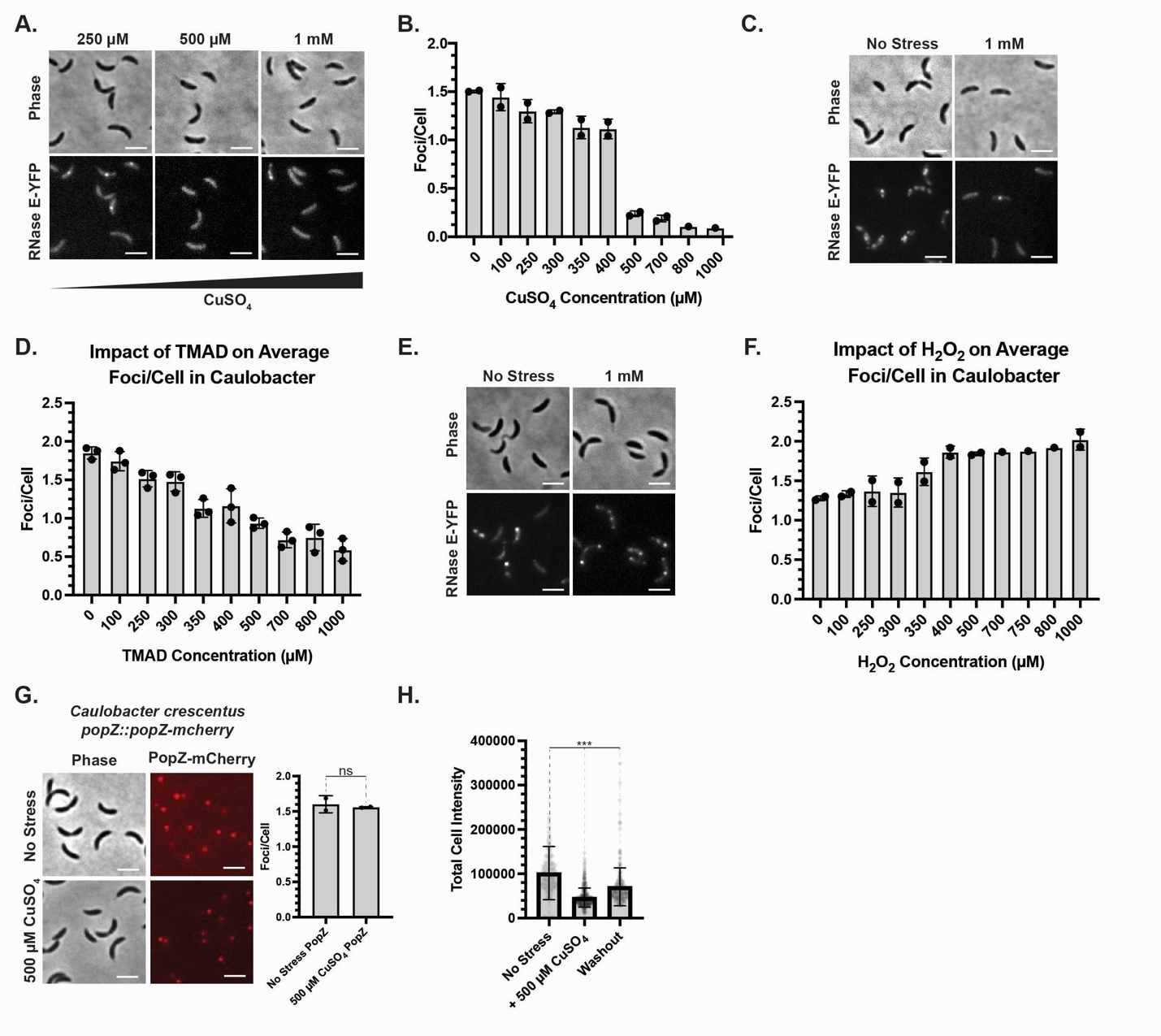


**SI Figure 1:** **Extensive *Caulobacter crescentus* BR-body dissolution is RNase E- and copper-specific with mild dissolution effects in the presence of TMAD and an increase in BR-bodies/cell in the presence of H_2_O_2_** (A) Representative phase contrast and fluorescence microscopy imaging of *C. crescentus* expressing RNase E-eYFP from its endogenous promoter in the presence of 250 µM CuSO_4_, 500 µM CuSO_4_, and 1 mM CuSO_4_ for 8 min. BR-body dissolution is promoted with increasing concentrations of CuSO_4_. The scale bar denotes 3 µm. (B) Quantification of the number of BR-bodies per cell in *C. crescentus* expressing RNase E-eYFP with varying concentrations of CuSO_4_ from 0-1000 µM. (C) Representative phase contrast and fluorescence microscopy imaging of *C. crescentus* expressing RNase E-eYFP from its endogenous promoter in the absence and presence of 1 mM TMAD for 8 min. BR-body dissolution is promoted with increasing concentrations of TMAD. The scale bar denotes 3 µm. (D) Quantification of the number of BR-bodies per cell in *C. crescentus* expressing RNase E-eYFP with varying concentrations of TMAD from 0-1000 µM. (E) Representative phase contrast and fluorescence microscopy imaging of *C. crescentus* expressing RNase E-eYFP from its endogenous promoter in the absence and presence of 1 mM H_2_O_2_ for 8 min. An increase in BR-bodies/cell is promoted with increasing concentrations of H_2_O_2_. The scale bar denotes 3 µm. (F) Quantification of the number of BR-bodies per cell in *C. crescentus* expressing RNase E-eYFP with varying concentrations of H_2_O_2_ from 0-1000 µM. (G) Phase contrast and fluorescence microscopy imaging of *C. crescentus* expressing PopZ-mCherry from its endogenous promoter in the absence and presence of 500 µM CuSO_4_. Quantitative analysis of *C. crescentus* expressing PopZ-mCherry from its endogenous promoter in the absence and presence of 500 µM CuSO_4_ (n=322,99 respectively). (H) Quantification of total cell intensity in arbitrary units corresponding to CuSO_4_ washout experiment with *C. crescentus* expressing RNase E-eYFP from its endogenous promoter (JS51).

**SI Figure 2: Broad spectrum and copper-specific chelators promote an increase in RNase E foci intensity and confirm the importance of phase separation for high fitness under metal chelation .** (A) Overlay of phase contrast and fluorescence microscopy imaging of a *C. crescentus* strain expressing the vanillate-inducible RNase E-eYFP (JS49, *vanA::rne-eYFP*) grown in modified HIGG media in the absence and presence of 1.6 mM CuSO_4_ over 1 hour. (B) Quantification of total cell intensity in arbitrary units, maximum intensity in arbitrary units, and the average number of BR-bodies per cell in the absence (gray) and presence (blue) of 1.6 mM CuSO_4_, respectively. BR-bodies were significantly more intense in cells grown under high Cu stress (high Cu: 1509±675 AU, n=1229; low Cu: 1097±472 AU, n=893, *p* < 0.0001). BR-body number was also significantly higher under high Cu stress (high Cu: 1.56±0.79, n=571; low Cu: 1.49±0.68, n=848, *p* = 0.0038). However, total cell intensity was also significantly different (high Cu 102350±38099 AU vs low Cu 82647±29702 AU, *p* = 0.0027). (C) Analysis of fluorescence intensity of BR-bodies represented by RNase E-YFP foci in *C.crescentus* *rne:rne-eyfp* foci when exposed to various metal chelators. Metal binding screen of broad spectrum EDTA metal chelator versus Bathocuproine sulfonate (BCS), Neocuproine (Nc) and Ammonium Tetrathiomolybdate (TTM). (D) Efficiency of Plating assay with wildtype *C. crescentus* RNase E, degradosome binding site mutant (ΔDBS) RNase E and NTD C-terminal deletion mutant (ΔCTD) RNase E in the absence and presence of EDTA and all copper chelators. BR-body phase separation provides enhanced fitness in the presence of broad-spectrum EDTA metal chelator and all copper-specific chelators. (E) Efficiency of Plating assay with wildtype *C. crescentus* RNase E, degradosome binding site mutant (ΔDBS) RNase E and NTD C-terminal deletion mutant (ΔCTD) RNase E in the absence and presence of 120 µM CuSO_4_, 130 µM CuSO_4_, 160 µM CuSO_4_, 200 µM CuSO_4_ and 250 µM CuSO_4_. (F) Efficiency of Plating assay with wildtype A*. tumefaciens C58*RNase E and the RNase e NTD C-terminal deletion mutant (ΔCTD) RNase E in the absence and presence of 3 mM CuSO_4_.

**
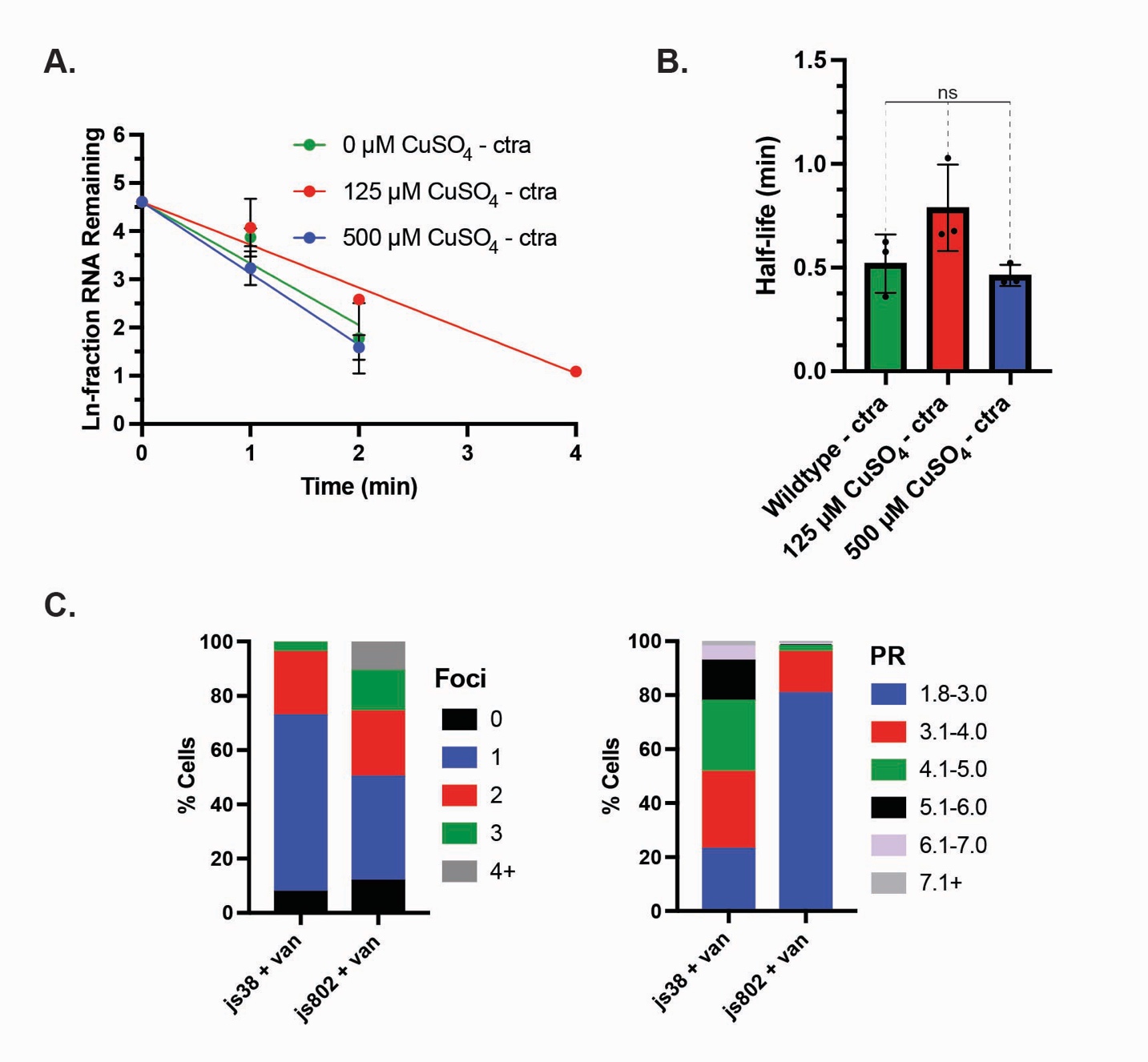
**

**SI Figure 3: Recruitment of RNase E clients into BR-bodies and BR-body phase separation provide enhanced fitness in the presence of increasing copper stress.** (A) Plot of Ln-fraction of RNA remaining for wild-type RNase E in the absence and presence of 125 µM CuSO_4_ and 500 µM CuSO_4_. (B) Quantification of mRNA half-life for wild-type RNase E in the absence and presence of 125 µM CuSO_4_ and 500 µM CuSO_4_ mRNA half-life decreases at elevated CuSO_4_ concentrations (*p>0.05* for all pairwise comparisons). (C) Quantification of the number of BR-bodies per cell and partition ratio for wildtype (js38 + van) and mutant RNase E C461A, C464A (js802 + van). The RNase E mutant variant displays more heterogeneous foci/cell.

**SI Figure 4: Polyphosphate partially co-localizes with BR-bodies in Caulobacter crescentus under no stress and low copper conditions. (A)** Representative phase contrast, RNase E-YFP, DAPI and merged stained polyphosphate granule fluorescence images of wild-type *C. crescentus* (*rne::rne-eyfp*) cells under control and copper stress conditions. The scale bar denotes 3 µM. **(B)** Co-localization analysis of BR-bodies and DAPI-stained polyphosphate granules shows that 67.24% of control cells under no stress conditions exhibit overlap between RNase E-YFP and polyphosphate foci. Pearson correlation analysis quantifying the spatial co-localization between RNase E-YFP and DAPI-stained polyphosphate granules reveals that copper stress does not significantly enhance their colocalization (6 biological replicates).

**
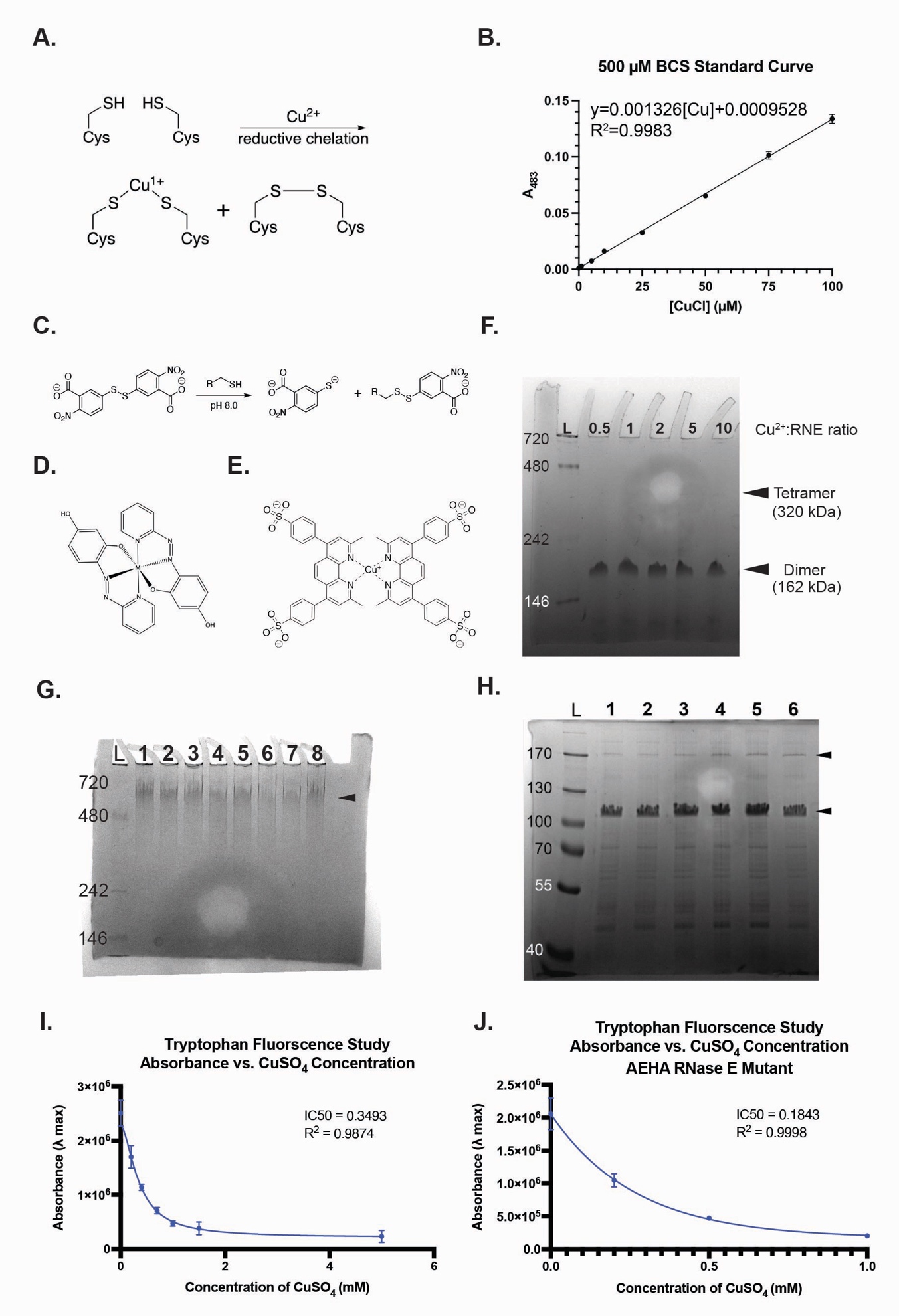
**

**SI Figure 5:** (A) Reductive chelation of Cu^2+^ by proximal cysteines. The proximal cysteines in RNase E could be oxidized by Cu^2+^ by reductive chelation. Cu^2+^ can interact with proximal RNase E cysteines, oxidizing one set of cysteines to cysteine and chelating the other as (Cys)_2_Cu^1+^. (B) Bathocuproine sulfonate standard curve with CuCl. Various concentrations of CuCl dissolved in 100 mM Tris-Cl pH 8.0 with 1 mM DTT was allowed to react with 500 µM BCS for 2 min prior to A_483_ measurement. The extinction coefficient was determined to be 13260 M^-1^cm^-1^ based on the slope of a fit linear regression. Error was based on three replicates. (C) Reaction scheme for Ellman Assay. (D) PAR reagent coordinating with a metal (M) either Cu^2+^ or Zn^2+^. (E) BCS compound coordinating with Cu^1+^. (F) Native PAGE analysis of RNase E (451-898) upon Cu^2+^ incubation. Oligomerization analysis of RNase E (451-898) resolved on an 8% native polyacrylamide gel. L: resolved NativeMark (Thermofisher) in kDa. Lanes 1-5: Analysis of 6 µM RNase E (451-898) incubated with (1) no metals, (2) 3 µM CuSO_44_, (3) 6 µM CuSO_4_, (4) 30 µM CuSO_4_, (5) 60 µM CuSO_4_. Triangles represent apparent molecular weights of an RNase E (451-898) dimer (*bottom*, 200 kDa) and predicted RNase E tetramer based on the dimer mass (*top*, 400 kDa). (G) Native PAGE analysis of RNase E (1-898, D403C) after Cu^2+^ incubation. Oligomerization analysis of RNase E (1-898, D403C) resolved on an 8% native polyacrylamide gel. L: resolved NativeMark (Thermofisher) in kDa. Lanes 1-8: Analysis of 6 µM RNase E (1-898, D403C) incubated with (1) no metals, (2) 0.5X CuSO_4_, (3) 1X CuSO_4_, (4) 2.5X CuSO_4_, (5) 5X CuSO_4_, (6) 10X CuSO_4_, (7) 10X ZnSO_4_, (8) 100X ZnSO_4_. Triangle represents the apparent mass of the protein dimer (625 kDa). (H) Non-reducing SDS-PAGE analysis of RNase E (451-898) upon Cu^2+^ incubation. Oligomerization analysis of RNase E (451-898) resolved on a 10% non-reducing SDS polyacrylamide gel. L: resolved PageRuler (Thermofisher) in kDa. Lanes 1-5: Analysis of 6 µM RNase E (451-898) incubated with (1) no metals, (2) 0.25X CuSO_4_, (3) 0.5X CuSO_4_, (4) 1X CuSO_4_, (5) 5X CuSO_4_, (6) 10X CuSO_4_. Triangles represent apparent molecular weights of an RNase E (451-898) dimer (bottom, 110 kDa) and predicted RNase E tetramer based on the dimer mass (top, 220 kDa). (I) Absorbance vs. CuSO_4_ concentration for unlabeled RNase E (451-898) (J) Absorbance vs. CuSO_4_ concentration for C461A, C464A unlabeled RNase E (451-898) variant. Titration by Cu(II) monitored by protein intrinsic fluorescence intensity quenching. Increasing concentrations of CuSO_4_ stress result in a decrease in tryptophan fluorescence intensity.


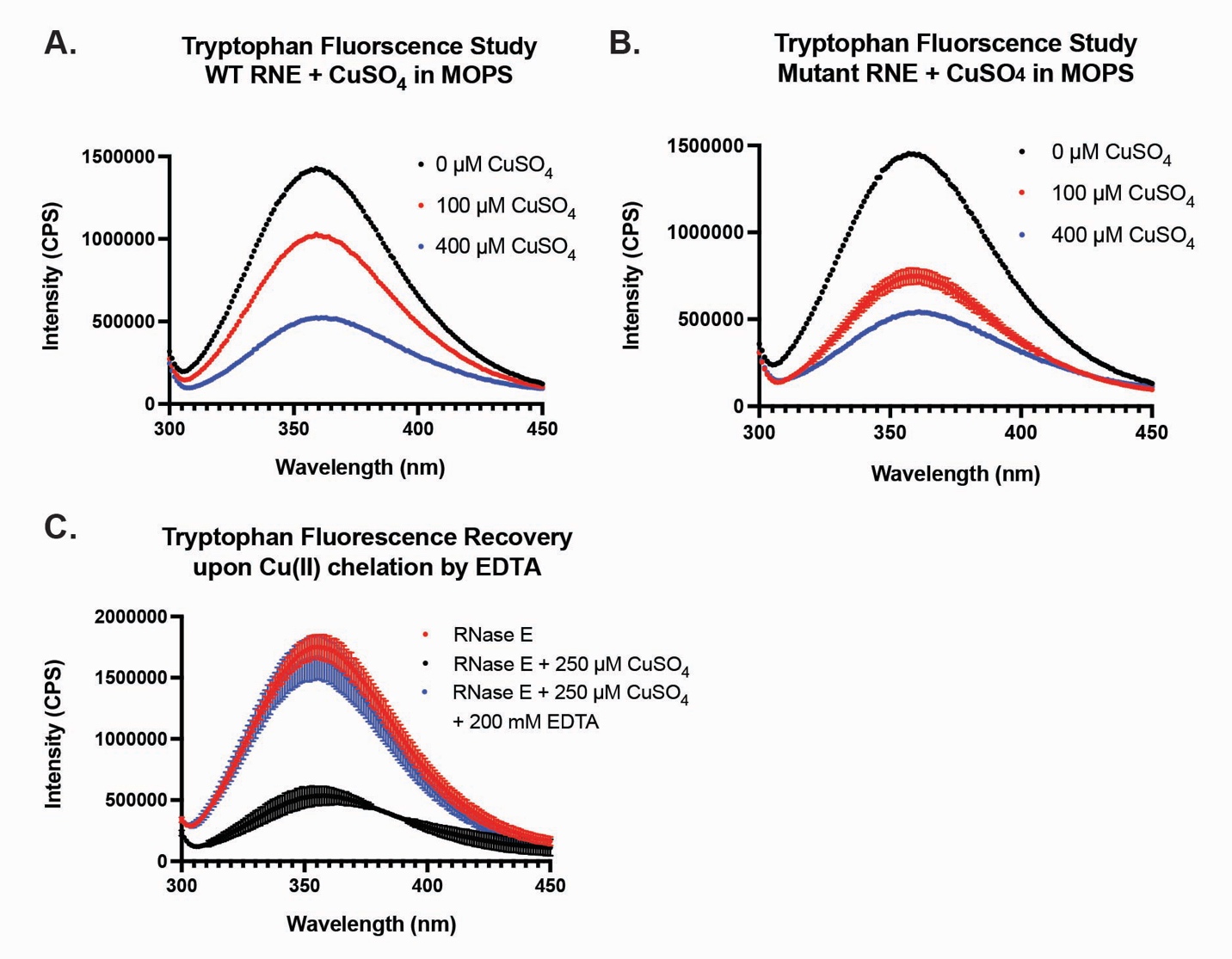


**SI Figure 6:** (A) Unlabeled wildtype RNase E (451-898) titration by Cu(II) in MOPS buffer monitored by protein intrinsic fluorescence intensity quenching. Increasing concentrations of CuSO_4_ stress result in a decrease in tryptophan fluorescence intensity. (B) Unlabeled mutant RNase E C461A/C464A titration by Cu(II) in MOPS buffer monitored by protein intrinsic fluorescence intensity quenching. Increasing concentrations of CuSO_4_ stress result in a decrease in tryptophan fluorescence intensity. (C) Tryptophan fluorescence recovery upon Cu(II) chelation by EDTA.

**
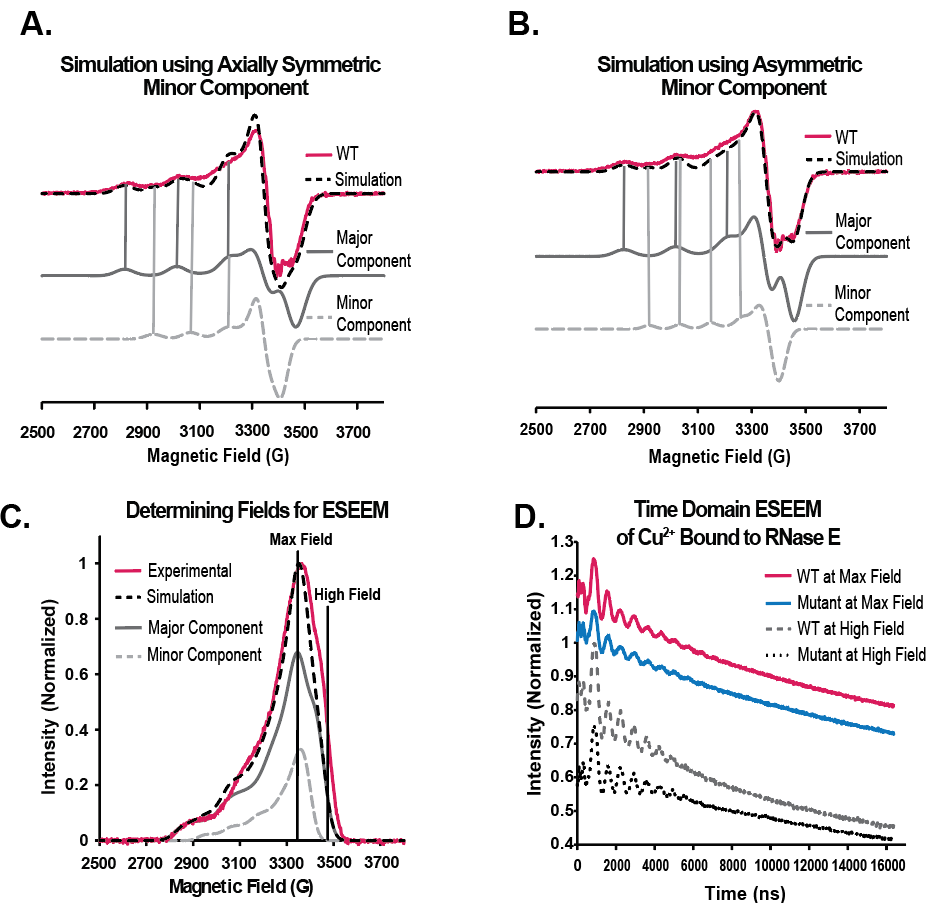
**

**SI Figure 7:** Simulation of RNase E wild-type (pink) is overlaid in dashed black. The RNase E spectrum shows two components. The major component (grey) accounts for 75% of the simulation (dashed black), and the minor component (dashed light grey) accounts for the remaining 25% of the simulation (dashed black). (A) Simulation of RNase E wild-type fit with an axially symmetric minor component (light grey). (B) Simulation of RNase E wild-type fit with an asymmetric minor component (light grey). (C) Field swept spectrum of the RNase E wild-type (pink) overlaid with the integrated CW-EPR simulation (dashed black) using an asymmetric coordination geometry for the minor component (dashed light grey). ESEEM experiments were carried out at the field with the maximum intensity (3340 G) and a higher field (3480 G), highlighted with black lines. At the high field, only the major component (grey) contributes to the spectrum. (D) Raw time domain ESEEM signal collected at the maximum intensity and higher field. Data has been normalized and offset for clarity.

**
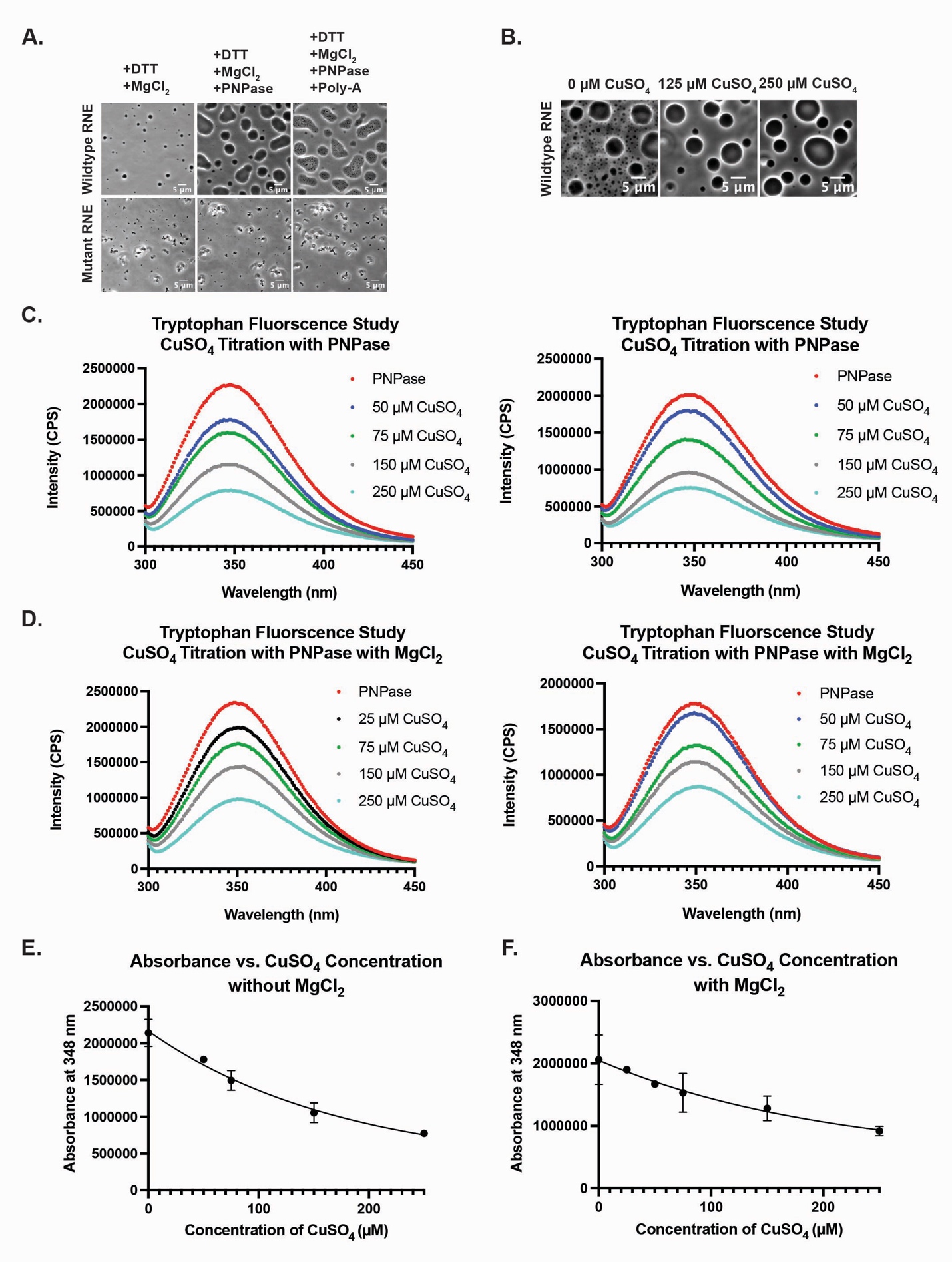
**

**SI Figure 8:** (A) The addition of PNPase increases RNase E droplet size, enhancing RNase E’s ability to sequester Poly-A into RNase E condensates, as can be visualized by the presence of dark speckles within RNase E-PNPase droplets. In comparison, in the presence of PNPase and poly(A), the RNase E (451-898) C461A/C464A variant forms aggregate-like assemblies. Samples were incubated for 1 hour prior to imaging. (B) RNase E droplets maintain spherical morphology in the presence of PNPase with increasing CuSO_4_ concentration. (C) Unlabeled PNPase titrations (replicates) by Cu(II) monitored by protein intrinsic fluorescence intensity quenching. Increasing concentrations of CuSO_4_ stress result in a decrease in tryptophan fluorescence intensity. (D) Unlabeled PNPase titration by Cu(II) in the presence of MgCl_2_ (replicates) monitored by protein intrinsic fluorescence intensity quenching. Increasing concentrations of CuSO_4_ stress result in a decrease in tryptophan fluorescence intensity. (E) Absorbance vs. CuSO_4_ concentration for Unlabeled PNPase in the absence of MgCl_2_ (F) Absorbance vs. CuSO_4_ concentration for Unlabeled PNPase in the presence of MgCl_2_.


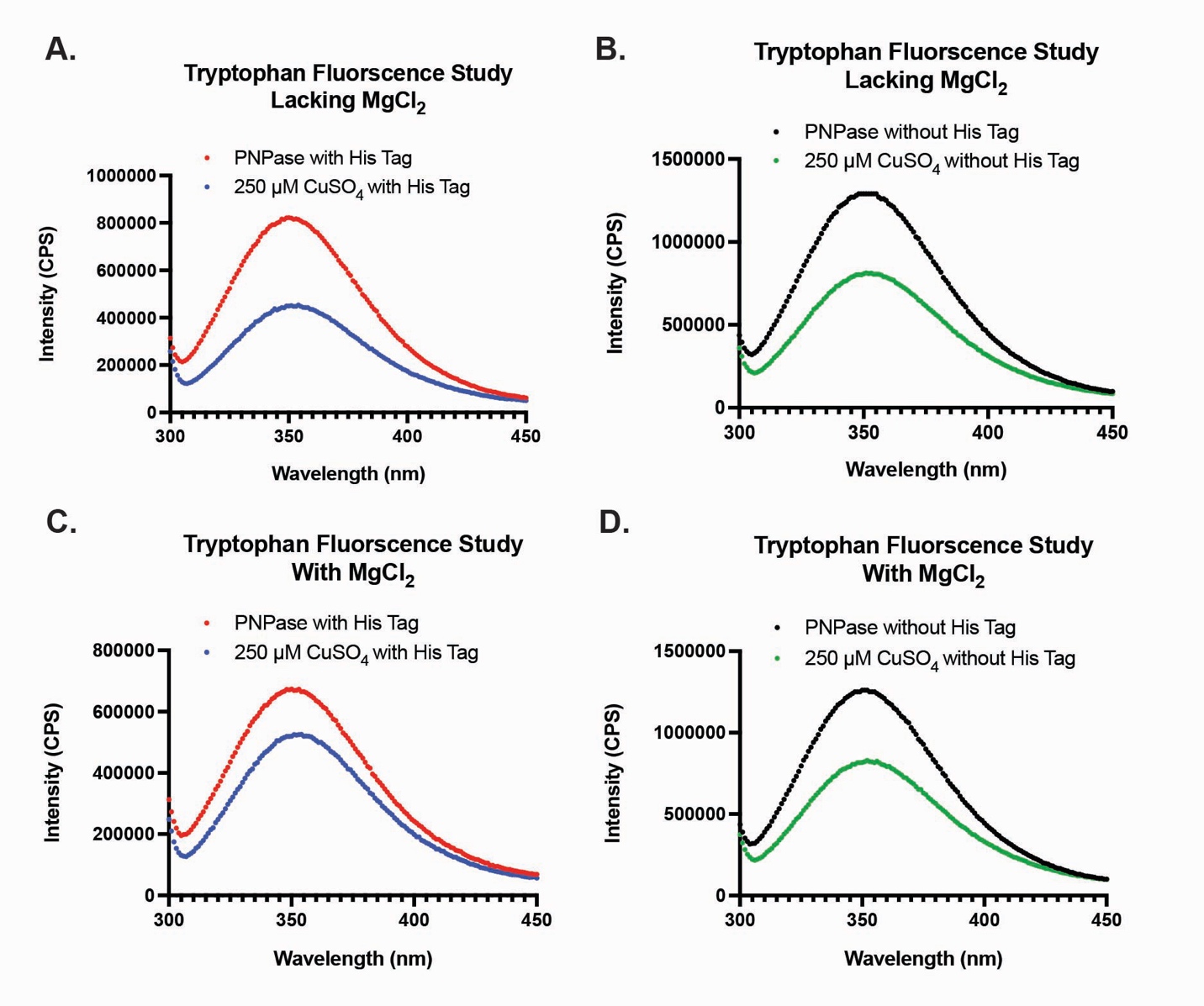


**SI Figure 9:** (A) Unlabeled PNPase with a histidine tag titration by Cu(II) in the absence of MgCl_2_ monitored by protein intrinsic fluorescence intensity quenching. Increasing concentrations of CuSO_4_ stress result in a decrease in tryptophan fluorescence intensity. (B) Unlabeled PNPase without a histidine tag titration by Cu(II) in the absence of MgCl_2_ monitored by protein intrinsic fluorescence intensity quenching. Increasing concentrations of CuSO_4_ stress result in a decrease in tryptophan fluorescence intensity. (C) Unlabeled PNPase with a histidine tag titration by Cu(II) in the presence of MgCl_2_ monitored by protein intrinsic fluorescence intensity quenching. Increasing concentrations of CuSO_4_ stress result in a decrease in tryptophan fluorescence intensity. (D) Unlabeled PNPase without a histidine tag titration by Cu(II) in the presence of MgCl_2_ monitored by protein intrinsic fluorescence intensity quenching. Increasing concentrations of CuSO_4_ stress result in a decrease in tryptophan fluorescence intensity.

**Table S1.** A summary of g and hyperfine tensors for Cu^2+^ in RNase E samples was obtained via simulations using EasySpin.

| Sample | Major Component | | | | Axially Symmetric Minor Component | | | | | | | | |
| --- | --- | --- | --- | --- | --- | --- | --- | --- | --- | --- | --- | --- | --- |
|  | g_║_ | A_║_ (G) | g_┴_ | A_┴_ (G) | g_║_ | | A_║_ (G) | | | g_┴_ | | A_┴_ (G) | |
| WT | 2.218 | 187 | 2.058 | 3 | 2.203 | | 136 | | | 2.062 | | 3 | |
| Mutant | 2.218 | 187 | 2.058 | 3 | 2.203 | | 136 | | | 2.062 | | 3 | |
| CuSO_4_ in HEPES | 2.270 | 161 | 2.053 | 5 | N/A | | | | | | | | |
|  | Major Component | | | | Asymmetric Minor Component | | | | | | | | |
|  | g_║_ | A_║_ (G) | g_┴_ | A_┴_ (G) | g_zz_ | A_zz_ (G) | | g_yy_ | A_yy_ (G) | | g_xx_ | | A_xx_ (G) |
| WT | 2.218 | 187 | 2.058 | 3 | 2.233 | 112 | | 2.058 | 7 | | 2.040 | | 11 |
| Mutant | 2.218 | 187 | 2.058 | 3 | 2.233 | 112 | | 2.058 | 7 | | 2.040 | | 11 |

**Table S2 Plasmid Construction Table**

| **Plasmid Name** | **Description** | **Vector** | **Digest Site** | **Template** | **Sense Primer** | **Antisense Primer** |
| --- | --- | --- | --- | --- | --- | --- |
| pDT058 | *pET28::rne*(451-898)-YFP | pET28 | -- | -- | -- | -- |
| pDT060 | pET28-RNase E(1-898) | pET28 | -- | -- | -- | -- |
| pDT074 | RNase E (451-898) | pTEV5 | NheI | pDT058 | DT061 | DT085 |
| pDT124 | RNase E (451-898, C461A, C464A)-eYFP | pTEV5 | NheI | pDT058 | DT061 | DT168 |
|  |  |  |  | pDT058 | DT167 | DT123 |
| pDT156 | RNase E (451-898, C464A)-eYFP | pTEV5 | NheI | pDT058 | DT061 | DT203 |
|  |  |  |  | pDT058 | DT202 | DT123 |
| pDT157 | RNase E (451-898, C461A)-eYFP | pTEV5 | NheI | pDT058 | DT061 | DT204 |
|  |  |  |  | pDT058 | DT205 | DT123 |
| pDT280 | MBP-RNase E (1-898, D403C) | pTEV5 | NheI | pTEV6 | DT471 | DT472 |
|  |  |  |  | pDT060 | DT423 | DT275 |
|  |  |  |  | pDT060 | DT274 | DT085 |
| pMJC0094 | CcPNPase | pTEV5 | NheI | -- | -- | -- |
| pHY001 | *pNTPS-rne::rneΔDBS specR* | pNTPS-138 | -- | pv*RNE(ΔDBS)-YFP* GentR, and pNTPS-*rne::rneΔCTD specR* | HY16F, HY17F | HY16R, HY17R |
| pVYFPRNE-doublecysala-YFP | PvanA::rne(1-898, C461A, C464A)-eYFP gentR | pvYFP-C4 | EcoR1, Nde1 | synthesized | -- | -- |
| patNTD-YFP | prne*ΔCTD*-eYFP gentR | pYFPC-4 | NdeI/EcoR1 | 1. *tumefaciens* genome | agro_ntd-F | agro_ntd-R |

* For plasmid construction of strains by the Schrader Lab (JS51, JS38), please see Al-Husini *et al*. *Mol. Cell.* **2018**, *71*, 1027-1039.

**Table S3 Strains used in this study.**

| **Strain Name** | **Description** | **Organism** | **Source** |
| --- | --- | --- | --- |
| DH5α | Bacterial cloning strain | *E. coli* | Invitrogen |
| Top10 | Bacterial cloning strain | *E. coli* | Invitrogen |
| BL21 (DE3) | Bacterial expression strain | *E. coli* | Novagen |
| Rosetta™ (DE3) | Bacterial expression strain | *E. coli* | Novagen |
| NA1000 | Synchronizable version of CB15 | *C. crescentus* | Shapiro Lab |
| DTT008 | pDT058 | BL21 (DE3) | Dylan’s Thesis/Childers Lab |
| DTT028 | pDT074 | Rosetta™ (DE3) | Dylan’s Thesis/Childers Lab |
| JS38 | *vanA::rne(1-577)-YFP* | NA1000 | Al-Husini et al Mol Cell 2018 |
| JS51 | *rne::rne(1-898)-YFP* | NA1000 | Al-Husini et al Mol Cell 2018 |
| WSC1748 | ParB::CFP-ParB; PopZ::mcherry-PopZ | Caulobacter with plasmid | This work/Childers Lab |
|  |  | NA1000 |  |
| DTT248 | PvanA::RNase E(1-898, C461A, C464A)-eYFP, Prne::pXrnessraC | *C. crescentus* | This work/Schrader Lab |
| JS802 |  | NA1000 |  |
| DTT249 | PvanA::RNase E(1-898)-eYFP, Prne::pXrnessraC | *C. crescentus* | Al-Husini et al Mol Cell 2018 |
| JS38 |  | NA1000 |  |
| JS495 | PvanA::RNase E(1-898, C461A, C464A)-eYFP | *C. crescentus* | This Work/Schrader Lab |
| MJC192 | PNPase | *C. crescentus* | This work/Childers Lab |
| JS5 | rne::rne-eYFP | *Agrobacterium tumefaciens* | Al-husini et al Mol cell 2018 |
| JS376 | rne::rneΔCTD-eYFP | *Agrobacterium tumefaciens* | This work/Schrader Lab |
| JS769 | rne::rneΔCTD | *C. crescentus* | Ortiz Rodriguez et al. |
| JS801 | rne::rneΔDBS | *C. crescentus* | This work/Schrader Lab |

**Table S4 Reagents**

# Reagents

| REAGENT or RESOURCE | SOURCE | IDENTIFIER |  |  |
| --- | --- | --- | --- | --- |
| Chemicals, peptides, and recombinant proteins |  |  |  |  |
| Gibson Master Mix  Copper (II) sulfate pentahydrate  TRizol Reagent  Spectinomycin  Rifampicin  Kanamycin  Nalidixic Acid  Sucrose  Phusion DNA polymerase  RNAprotect Bacterial Reagent  Luna® Universal One-Step RT-qPCR Kit  Qubit RNA HS Assay Kit  Chloroform  Glycogen, RNA grade  2-PROPANOL, ANHYDROUS  ETHANOL-D6 (D, 99%), ANHYD.  EDTA (0.5 M), pH 8.0, RNase-free  Tris (1 M), pH 7.0, RNase-free  Agar  Bactopeptone  Yeast extract  UltraPure™ Ethidium Bromide, 10 mg/mL  Thermo Scientific™ TriTrack DNA Loading Dye (6X)  Magnesium sulfate  Calcium chloride (97%)  Luria Broth Base  FD Dpn1  T4 DNA Ligase  Agarose  RNAprotect Bacteria Reagent  Gentamycin Sulfate  Vanillic acid  D(+)-Xylose, 99+% | New England BioLabs Inc.  Sigmaaldrich  15596018  Sigmaaldrich  Sigmaaldrich  Sigmaaldrich  Sigmaaldrich  sigmaaldrich  Thermo Scientific  QIAGEN  NEB  Thermo Fischer Scientific  Thermofisher scientific  Thermofisher scientific  Sigmaaldrich  Sigmaaldrich  Sigmaaldrich  Thermofisher scientific  Thermofisher scientific  Fisherchemicals  Thermofisher scientific  sigmaaldrich  Thermofisher scientific  Thermofisher scientific  sigmaaldrich  sigmaaldrich  sigmaaldrich  Thermofisher scientific  Thermofisher scientific  Qiagen  Sigmaaldrich  Sigmaaldrich  Thermofisher scientific | E2611S  7758-99-8  Ambion  S6501-25G  R7382-1G  K1377-5G  N8878-5G  S5016-25G  F-530L  76506  E3005L  Q32851  AC423550010  RO551  [67-63-0](https://www.sigmaaldrich.com/US/en/search/67-63-0?focus=products&page=1&perpage=30&sort=relevance&term=67-63-0&type=cas_number)  [1516-08-1](https://www.sigmaaldrich.com/US/en/search/1516-08-1?focus=products&page=1&perpage=30&sort=relevance&term=1516-08-1&type=cas_number)  AM9261  AM9851  DF0001-17-0  211677  92144-500G-F  15585011  FERR1161  M7506-1KG  746495-500G  12795084  ER1701  EL0011  A7705  76506  345814-1GM  H36001-25G  141005000 |  |  |
|  |  |  |  |  |
|  |  |  |  |  |
|  |  |  |  |  |
|  | | |  |  |
|  |  |  |  |  |

**Author contributions:**

H.Y. has generated *JS801* and *JS802* and conducted all mRNA half-life measurements.

J.G. has helped with the screening and generation of *JS801*. AG has generated strain JS495.
